# Synergistic Remodeling of Tumor Immune Microenvironment via a DNA Nanodevice Integrating STING Activation and Lysosome‐Targeted PD‐L1 Degradation

**DOI:** 10.1002/advs.75855

**Published:** 2026-05-27

**Authors:** Haoxiang Li, Min Hou, Shasha Sun, Jun Cao, Jian‐Hui Jiang, Jianjun He

**Affiliations:** ^1^ School of Biomedical Sciences State Key Laboratory of Chemo/Bio‐Sensing and Chemometrics College of Chemistry and Chemical Engineering Hunan University Changsha China; ^2^ School of Physics and Chemistry Hunan First Normal University Changsha China; ^3^ FuRong Laboratory Changsha China

**Keywords:** CGAS‐STING, immunotherapy, targeted protein degradation, tumor microenvironment, tumor treatment

## Abstract

The cGAS‐STING pathway is a cornerstone of innate antitumor immunity; however, its therapeutic potential is often limited by the inefficient cytosolic delivery of agonists and the immunosuppressive tumor microenvironment (TME). Here, we report a DNA nanostructure‐based platform that simultaneously induces lysosomal degradation of immune checkpoint protein PD‐L1 and activates the STING pathway. We demonstrate that STING activation further enhances PD‐L1 degradation, resulting in strong synergistic immune activation. In murine models of triple‐negative breast cancer, the DNA nanodevice effectively reprograms the TME by enhancing dendritic cell maturation and CD8^+^ T cell infiltration, resulting in potent suppression of both primary tumor growth and pulmonary metastases. This study establishes a versatile nanoplatform that bridges targeted protein degradation with STING‐mediated innate immune activation, provides a promise strategy for cancer immunotherapy.

## Graphical abstract

A rational design DNA nanoplatform not only achieves efficient PD‐L1 degradation but also triggers robust STING signaling. The nanodevice effectively reprograms “cold” tumors, leading to potent inhibition of tumor growth and metastasis in vivo.

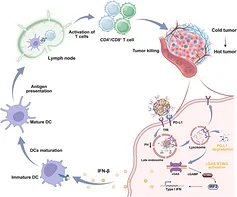

## 1 Introduction

The innate immune system constitutes the first line of defense against cellular anomalies, and the cyclic GMP‐AMP synthase (cGAS)‐stimulator of interferon genes (STING) pathway has emerged as a cornerstone mechanism for sensing cytosolic double‐stranded DNA (dsDNA), a hallmark of genomic instability, viral infection, and cellular stress [1]. STING activation triggers the release of type I interferons and inflammatory cytokines, thereby promoting dendritic cell maturation, enhancing antigen presentation, activating T cells, and remodeling the tumor immune microenvironment to elicit antitumor immunity [2, 3]. Given its pivotal role in immunosurveillance, the cGAS‐STING pathway has been intensively pursued as a therapeutic target, with several STING agonists advancing to clinical trials [4, 5, 6].

Nevertheless, two major challenges continue to hinder the clinical development of STING‐based therapies. First, conventional STING agonists, including cyclic dinucleotides and recently developed small‐molecule mimetics (e.g., diABZI), often suffer from poor pharmacokinetic profiles, rapid systemic clearance, and limited tumor selectivity [6, 7]. Second, the efficacy of STING monotherapy is frequently compromised by the highly immunosuppressive tumor microenvironment (TME), particularly in “cold” tumors characterized by poor T cell infiltration [8, 9, 10]. Consequently, current cGAS–STING therapies are often combined with antibodies targeting immune checkpoint proteins such as programmed cell death ligand 1 (PD‐L1) [11, 12, 13]. However, such combinatorial approaches increase safety risks and pharmacokinetic complexity [14, 15]. Therefore, strategies enabling precise delivery and rational integration of STING activation with immune modulation are crucial for advancing cGAS–STING therapeutics.

Recent efforts have sought to combine cGAS‐STING activation with PD‐L1 inhibition to achieve enhanced therapeutic outcomes [16, 17, 18, 19]. For instance, Wang and colleagues developed a carbon‐dot‐based PROTAC (CDTACs) for STING activation and PD‐L1 degradation [17], though its cellular internalization required additional fasting‐mimicking diet interventions. Mi et al. recently reported a nanoparticle co‐encapsulating 2,3‐cGAMP and PD‐L1 siRNA, which demonstrated strong antitumor effects in vivo [16]. Meanwhile, a variety of nanoparticle‐based strategies have been developed to activate the STING pathway [20, 21, 22]. However, such approaches often involve complex synthesis and carry potential off‐target risks associated with siRNA. Noticeably, most existing approaches rely on STING agonists or metal ions to activate the STING pathway [18, 23], whereas natural cGAS–STING signaling is triggered by short dsDNA [24, 25].

**SCHEME 1:**
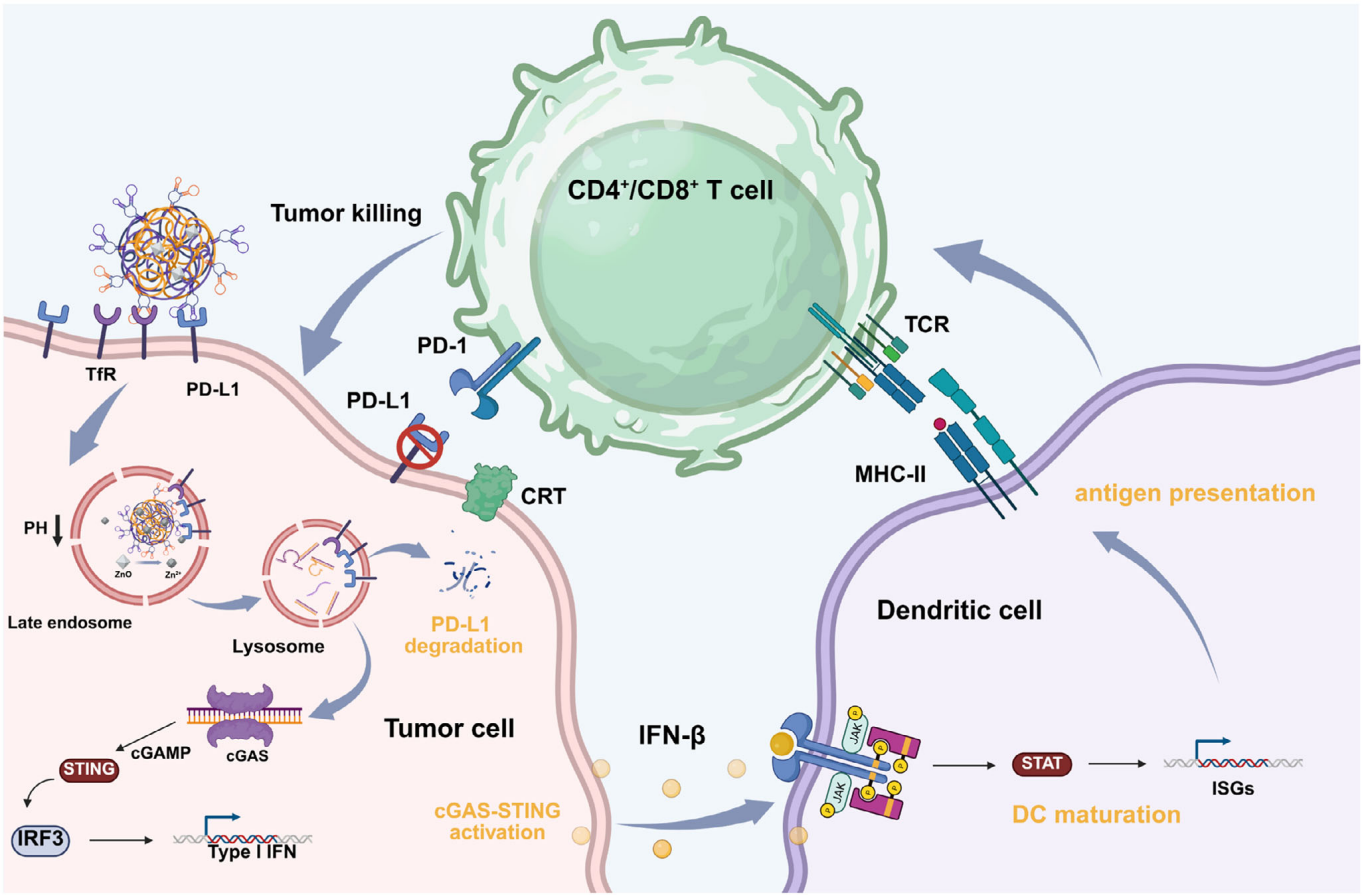
Schematic illustration of TfR/PD‐L1 bispecific DNA nanostructure that hijack lysosome‐associated TfR to specifically degrade PD‐L1 and activate the cGAS‐STING pathway. Upon TfR‐mediated internalization, targeted PD‐L1 underwent lysosomal degradation; while its DNA framework is cleaved by DNAzyme, releasing the cGAS‐binding sequence. Consequently, synergistic PD‐L1 degradation and cGAS–STING activation are achieved by TfR/PDL1‐GBS@ZnO, leading to dendritic cell maturation and subsequent T‐cell activation for tumor microenvironment remodeling. (Created in Biorender.com).

Here, we report the development of a DNA nanodevice‐based platform that activates the cGAS–STING pathway while equipping lysosome‐targeting aptamer chimeras (STTAC) for down‐regulating PD‐L1 (Scheme 1). We employed aptamers hijacking tumor‐specific lysosome‐associated transferrin receptor (TfR) [26, 27, 28] to specifically degrade PD‐L1. Additionally, to effectively activate cGAS‐STING, short dsDNA fragments [29] that activate cGAS are released through a Zn^2^^+^‐dependent DNAzyme (I‐R3) cleavage reaction [30], triggered by ZnO dissolution in the acidic lysosomal compartment. We demonstrate that this DNA nanodevice effectively reprograms the immunosuppressive TME, suppressing primary tumor growth and inhibiting metastasis in murine 4T1 breast cancer models. This work presents a DNA‐based therapeutic strategy with broad potential for cancer immunotherapy.

## 2 Results and Discussion

### 2.1 Design and Synthesis of DNA Nanodevice‐Based STTAC

**FIGURE 1:**
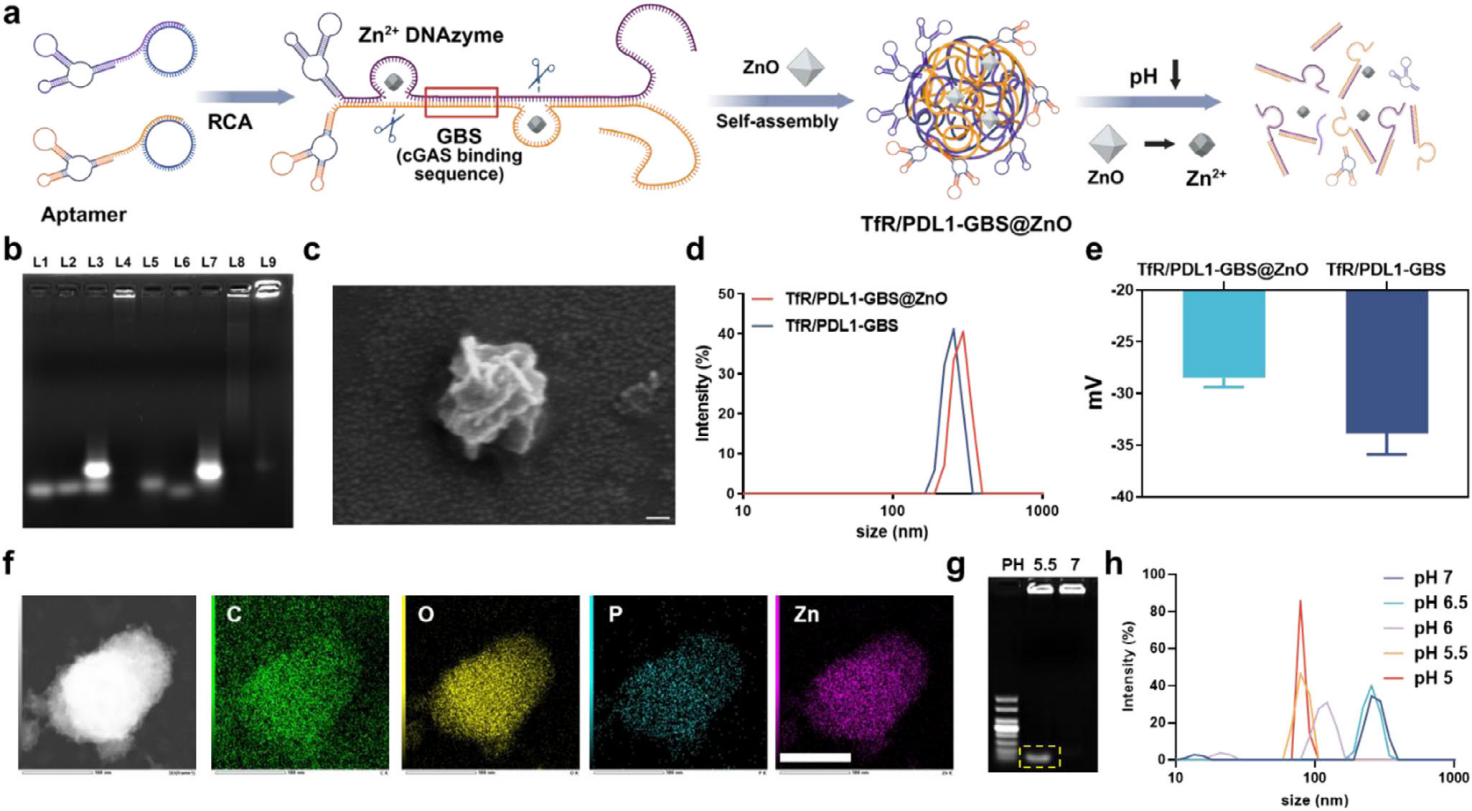
Preparation and characterization of TfR/PDL1‐GBS@ZnO. (a) Schematic illustration of TfR/PDL1‐GBS@ZnO assembly, Created in BioRender.com. (b) Agarose gel electrophoresis analysis of the TfR/PDL1‐GBS@ZnO, L1: Primer‐TfR, L2: Template‐TfR‐GBS, L3: Circle template‐TfR‐GBS, L4: R‐TfR‐GBS, L5: Primer‐PDL1, L6: Template‐PDL1‐GBS, L7: Circle template‐PDL1‐GBS, L8: R‐PDL1‐GBS, L9: TfR/PDL1‐GBS@ZnO. (c) SEM image of the TfR/PDL1‐GBS@ZnO, scale bar = 100 nm. (d) Size distribution of TfR/PDL1‐GBS and TfR/PDL1‐GBS@ZnO determined by DLS measurements. (e) Zeta potentials of TfR/PDL1‐GBS and TfR/PDL1‐GBS@ZnO. (f) TEM and EDS analyses of TfR/PDL1‐GBS@ZnO, scale bar = 100 nm. (g) Agarose gel electrophoresis analysis of Zn^2+^‐induced TfR/PDL1‐GBS@ZnO release under different pH. (h) Size distribution analysis of TfR/PDL1‐GBS@ZnO cleavage under different pH conditions.

To realize STTAC, we employed a dual rolling circle amplification (RCA) strategy to generate two polymeric DNA strands: one containing repetitive TfR aptamer, I‐R3 DNAzyme, and cGAS‐binding sequence (GBS) (R‐TfR‐GBS), and the other bearing PD‐L1 aptamer, I‐R3 DNAzyme, and GBS‐complementary sequences (R‐PDL1‐GBS). Self‐assembly of R‐TfR‐GBS and R‐PDL1‐GBS strands results in the formation of DNA nanoflower (TfR/PDL1‐GBS), and ZnO is further encapsulated into the DNA nanodevice (TfR/PDL1‐GBS@ZnO) to facilitate the DNAzyme‐mediated lysosomal release of short dsDNA that activates cGAS‐STING pathway (Figure 1a and Table S1).

Agarose gel electrophoresis analysis confirmed the formation of high‐molecular‐weight RCA products and the assembled nanodevice, which showed minimal migration on agarose gels (Figure 1b). Scanning electron microscopy (SEM) revealed the typical morphology of DNA nanoflowers (Figure 1c and Figure S1). The average hydrodynamic diameter of TfR/PDL1‐GBS@ZnO was ∼200 nm, with a narrow particle size distribution (100–300 nm) (Figure 1d). Encapsulation with ZnO raised the zeta potential of the DNA nanodevice from –33.8 to –28.5 mV (Figure 1e). And as confirmed by transmission electron microscopy (TEM) and energy‐dispersive X‐ray spectroscopy (EDS) analyses, ZnO was successfully encapsulated in the DNA nanodevice (Figure 1f and Figure S2). TfR/PDL1‐GBS@ZnO exhibited pH‐responsive cleavage under acidic conditions, as evidenced by a gradual decrease in size and the release of short DNA fragments (Figure 1g,h). Serum stability assays indicated that TfR/PDL1‐GBS@ZnO remained intact after 24 h in 10% FBS, whereas linear aptamer controls degraded within 12 h (Figure S3).

### 2.2 TfR‐Mediated Internalization of TfR/PDL1‐GBS@ZnO

**FIGURE 2:**
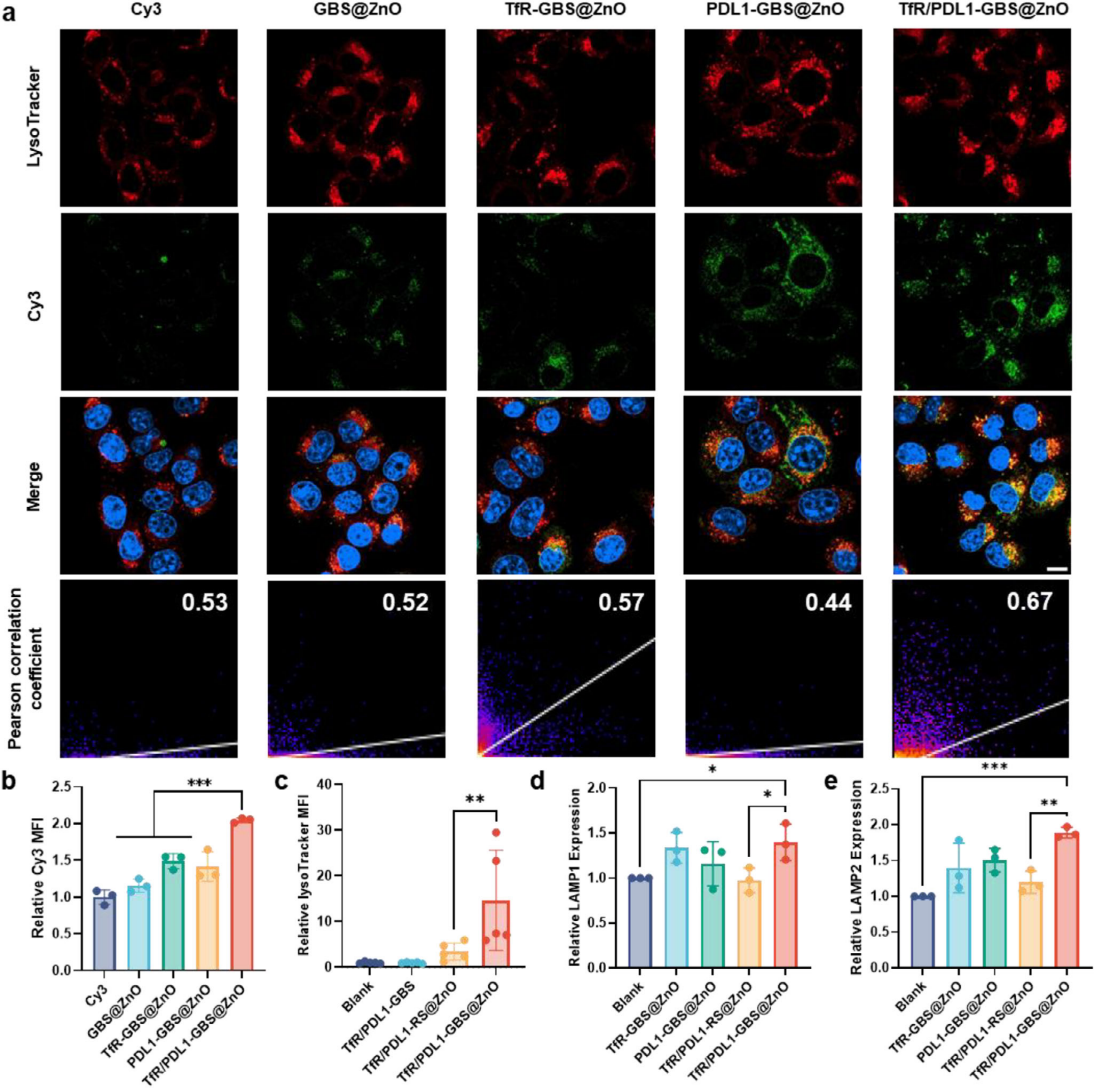
TfR‐mediated endocytosis and lysosomal trafficking. (a) Confocal fluorescence imaging analysis the cellular uptake and lysosomal colocalization of different DNA nanodevices in 4T1 cells. Scale bar = 10 µm. (b) Flow cytometry analysis of different DNA nanodevices endocytosis in 4T1 cells, *n* = 3. (c) Statistical analysis of MFI of LysoTracker treated with different groups, *n* = 5. Western blot analysis of relative LAMP1 (d) and LAMP2 (e) expression treated with different groups, *n* = 3. Data are presented as mean ± S.D., Significance (one‐way ANOVA) is labeled above the data, ^*^ *p* < 0.05, ^**^ *p* < 0.01, ^***^ *p* < 0.001.

We first verified the cellular internalization of TfR/PDL1‐GBS@ZnO in 4T1 cells, a metastatic mouse breast cancer cell line, which overexpressed both TfR and PD‐L1 [19]. Using Cy3‐conjugated TfR/PDL1‐GBS@ZnO, we observed efficient internalization into 4T1 cells, colocalizing with LysoTracker Red (Figure 2a). Meanwhile, medium intracellular fluorescence signals were observed for both the PD‐L1 aptamer–armed nanodevice (PDL1‐GBS@ZnO) and the TfR aptamer–armed nanodevice (TfR‐GBS@ZnO) when administered individually. Flow cytometry further confirmed enhanced internalization from TfR/PDL1‐GBS@ZnO (Figure 2b). These results indicated that TfR and PD‐L1 are both essential for the internalization of STTAC, and TfR/PD‐L1 bispecific targeting further enhances the active endocytosis process.

Additionally, we investigated the concentration dependence of TfR/PDL1‐GBS@ZnO‐mediated endocytosis. Increasing the TfR/PDL1‐GBS@ZnO concentration correspondingly increased the intracellular Cy3 signal (Figure S4). We then examined the time course of nanodevice internalization, and Cy3 fluorescence was detectable in lysosomes as early as 1 h after treatment (Figure S5). Recent studies have reported that activation of the cGAS‐STING pathway enhances lysosomal degradation efficiency by promoting the biogenesis of lysosomes and autophagic vesicles [31]. To validate this effect in our system, we prepared two control DNA nanodevices: one with randomly scrambled cGAS‐binding sequences (TfR/PDL1‐RS@ZnO) and one without ZnO encapsulation (TfR/PDL1‐GBS). As shown in Figure 2c, 4T1 cells treated with TfR/PDL1‐GBS@ZnO after 6 h exhibited a substantial increase in LysoTracker fluorescence intensity, suggesting that the effective release of short GBS is essential for enhancing lysosome activity. Furthermore, we applied western blotting (WB) to quantify the expression of lysosomal markers lysosomal‐associated membrane proteins (LAMP1 and LAMP2) and found significantly elevated LAMP1 and LAMP2 levels in TfR/PDL1‐GBS@ZnO compared to TfR/PDL1‐RS@ZnO group (Figure 2d,e and Figure S6). We further examined the expression of key autophagy‐related proteins, including TFEB, p62, and LC3 (Figure S7). The results suggest an increase in autophagic flux, which is consistent with previously reported mechanisms in which STING activation enhances lysosomal function [31]. These results support that cGAS‐STING activation increases lysosome activity.

### 2.3 TfR/PDL1‐GBS@ZnO‐Mediated Degradation of PD‐L1

**FIGURE 3:**
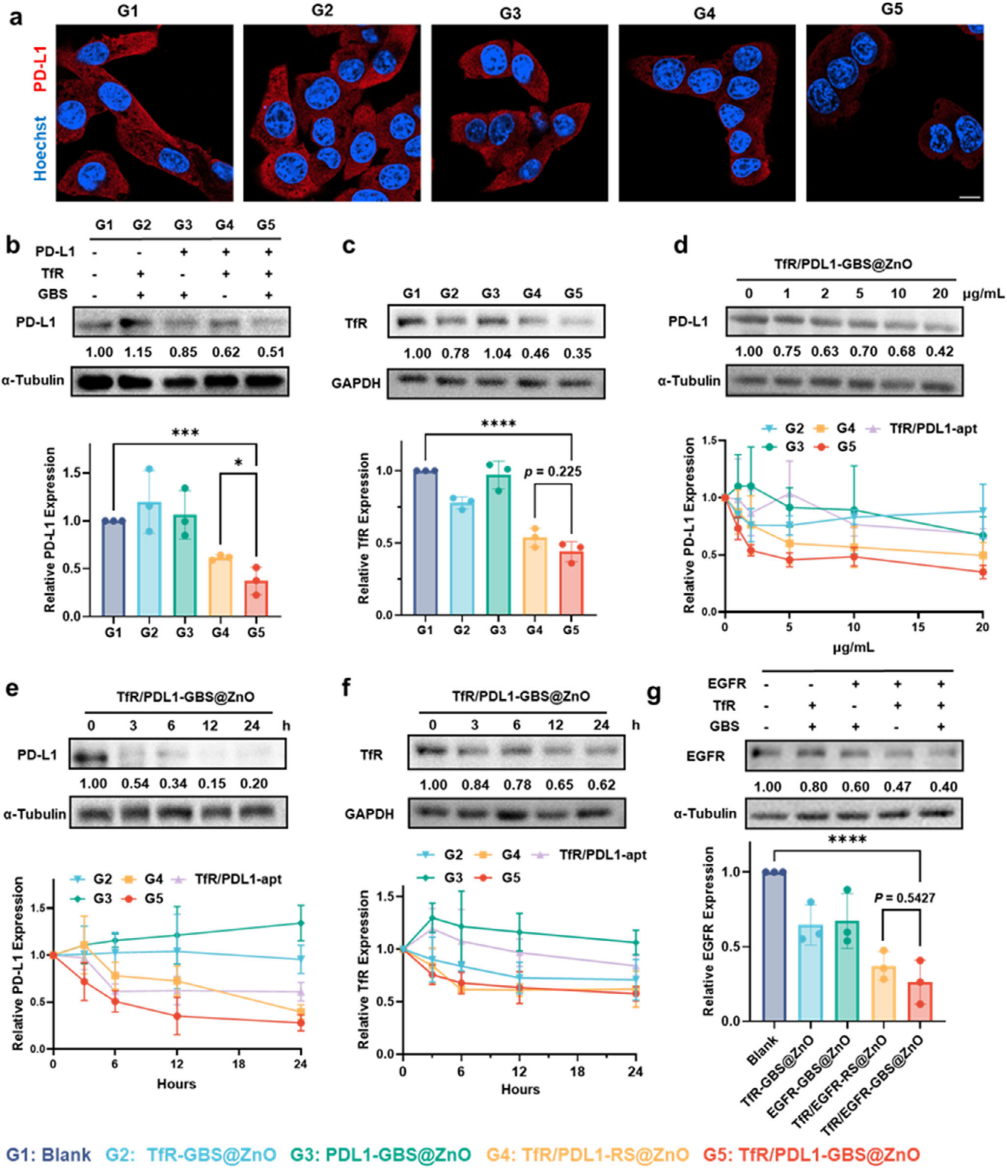
TfR/PDL1‐GBS@ZnO‐mediated membrane protein degradation. (a) Immunofluorescence analysis of PD‐L1 degradation in 4T1 cells after different treatments. Scale bar = 10 µm. (b) Western blot and statistical quantification of PD‐L1 degradation after 6 h treatment with different groups in 4T1 cells. (c) Western blot and statistical analysis of relative TfR degradation after 6 h treatment treated with different groups. (d) Western blot and statistical quantification of relative PD‐L1 degradation after treatment with different DNA nanodevices at varying concentrations. (e) Western blot and statistical quantification of relative PD‐L1 degradation after treatment with different DNA nanodevices for the indicated times. (f) Western blot and statistical quantification of relative TfR degradation after treatment with different DNA nanodevices for the indicated times. (g) Western blot and statistical quantification of EGFR degradation after 6 h treatment with different groups in A549 cells. Data are presented as mean ± S.D., *n* = 3, Significance (one‐way ANOVA) is labeled above the data, ^*^ *p* < 0.05, ^***^ *p* < 0.001, ^****^ *p* < 0.0001.

We next evaluated the capacity of TfR/PDL1‐GBS@ZnO in degrading targeted proteins. Incubation of 4T1 cells with TfR/PDL1‐GBS@ZnO or TfR/PDL1‐RS@ZnO at 37°C for 6 h resulted in a marked reduction of PD‐L1 as compared to TfR‐GBS@ZnO and PDL1‐GBS@ZnO treatments (Figure 3a,b and Figures S8 and S9), confirming that TfR/PD‐L1 bispecific targeting is critical for inducing PD‐L1 degradation. TfR/PDL1‐GBS@ZnO treatment resulted in more efficient PD‐L1 degradation in 4T1 cells compared with TfR/PDL1‐RS@ZnO. In contrast, this enhanced degradation effect was not observed in the STING function‐deficient cell line DU145 (Figure S10) [32]. These results suggest that STING activation may contribute to the enhanced degradation of the target protein. Meanwhile, this TfR‐directed lysosomal sorting process also resulted in the reduction of TfR in TfR‐binding TfR‐GBS@ZnO, TfR/PDL1‐GBS@ZnO and TfR/PDL1‐RS@ZnO groups (Figure 3c), suggesting that multivalent TfR binding may compromise TfR recycling ability. Of note, the incorporation of GBS significantly enhanced the PD‐L1 degradation but not TfR. Furthermore, TfR/PDL1‐GBS exhibited weakened PD‐L1 degradation compared to TfR/PDL1‐GBS@ZnO (Figure S9). Taken together, these results suggested that activation of cGAS‐STING bolsters lysosomal degradation of PD‐L1. Dose–response analysis showed increasing degradation with higher TfR/PDL1‐GBS@ZnO concentrations, peaking at 20 µg/mL (Figure 3d), which was used for subsequent studies. Time‐course experiments revealed detectable PD‐L1 and TfR degradation within 3 h, reaching a maximum at 24 h (Figure 3e,f). We determined that the apparent half‐life of PD‐L1 following TfR/PDL1‐GBS@ZnO treatment was 2.79 h (Figure S11). Then, we compared TfR/PDL1‐GBS@ZnO with a reported TfR/PD‐L1 bispecific aptamer (TfR/PDL1‐apt) [15]. TfR/PDL1‐GBS@ZnO exhibited substantially greater PD‐L1 depletion than TfR/PDL1‐apt (Figures S5 and S12), suggesting multivalent binding enhanced targeted protein degradation. Lysosomal inhibitor chloroquine (CQ), but not proteasome inhibitor MG132 [33], abrogated the targeted protein degradation (Figure S13).

TfR/PDL1‐GBS@ZnO‐mediated PD‐L1 degradation was further validated in human triple‐negative breast cancer cells (MDA‐MB‐231) and Hela cells (Figure S14). To evaluate the general applicability, we prepared STTAC targeting TfR and epidermal growth factor receptor (EGFR) (TfR/EGFR‐GBS@ZnO) and found robust EGFR reduction in TfR/EGFR‐GBS@ZnO treated A549 cells (Figure 3g and Figure S15).

### 2.4 STTAC‐Mediated STING Pathway Activation and Dendritic Cell Maturation

**FIGURE 4:**
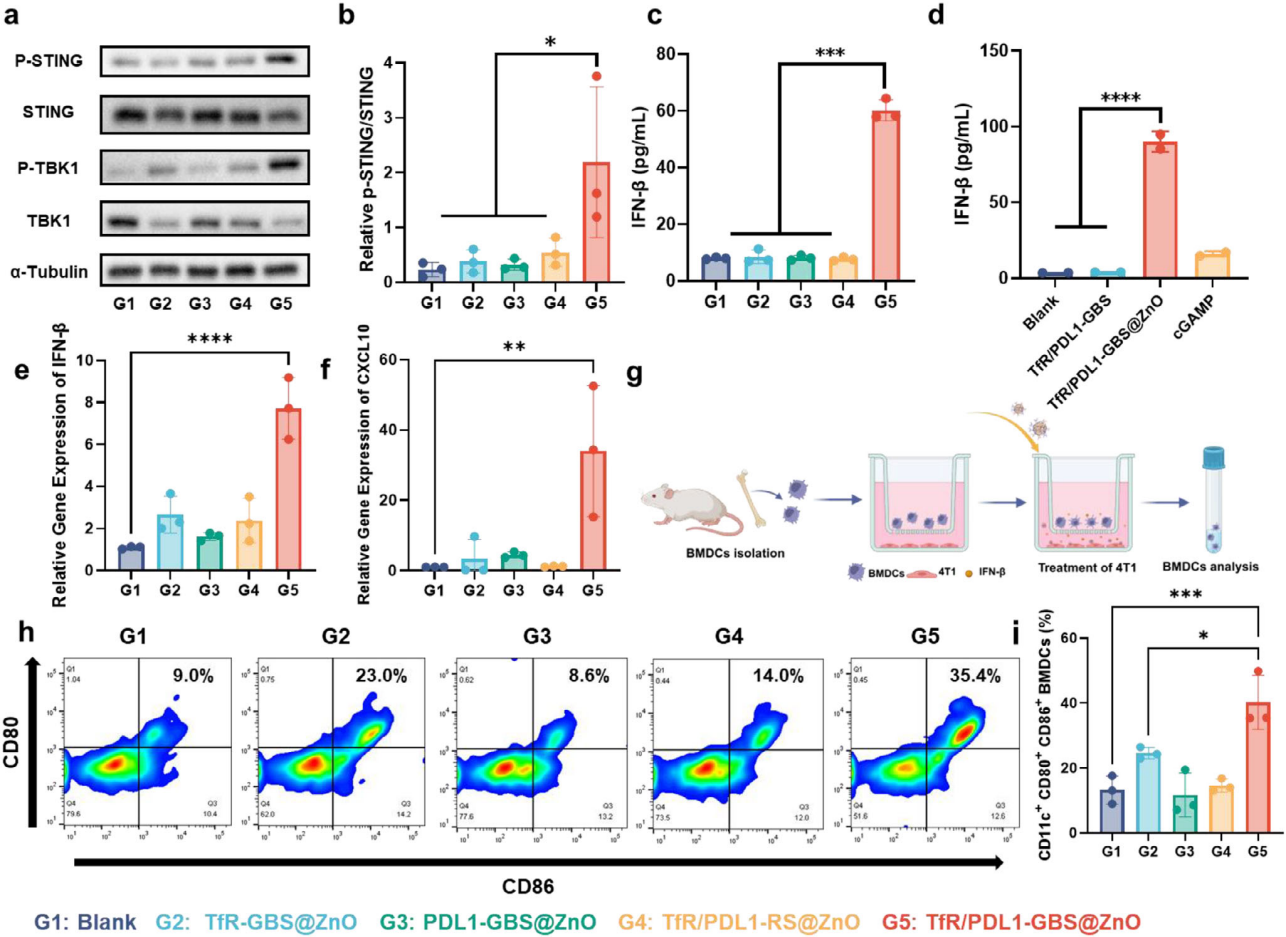
TfR/PDL1‐GBS@ZnO‐mediated STING activation and BMDCs maturation. (a) Western blot analysis of STING pathway activation and statistical analysis (b) of relative p‐STING/STING expression in 4T1 cells. (c) IFN‐β secretion treated with different groups. (d) IFN‐β secretion after treated with TfR/PDL1‐GBS, TfR/PDL1‐GBS@ZnO and cGAMP in 4T1 cells. mRNA expression of IFN‐β (e) and CXCL10 (f) treated with different groups. (g) Schematic illustration of in vitro assessment of BMDCs maturation. The upper layer is BMDCs and the lower layer is 4T1 cells. Created in BioRender.com. (h) Flow cytometry and statistical analysis i) of BMDCs maturation after different treatments. Data are presented as mean ± S.D., *n* = 3, Significance (one‐way ANOVA) is labeled above the data, ^*^ *p* < 0.05, ^**^ *p* < 0.01, ^***^ *p* < 0.001, ^****^ *p* < 0.0001.

To assess the ability of TfR/PDL1‐GBS@ZnO in activating cGAS‐STING pathway, we first examined phosphorylation of STING and its downstream effector TANK Binding Kinase 1 (TBK1) in 4T1 using WB [17]. TfR/PDL1‐GBS@ZnO treatment induced robust phosphorylation of both STING and TBK1, markedly exceeding that observed in all control groups (Figure 4a,b). Furthermore, significantly higher IFN‐β (a cytokine of cGAS‐STING pathway activation) secretion levels were detected in cells following TfR/PDL1‐GBS@ZnO treatment as compared to other groups (Figure 4c), demonstrating that TfR/PDL1‐GBS@ZnO‐mediated STING activation is strongly dependent on the incornporated GBS motif [29]. Moreover, TfR/PDL1‐GBS@ZnO elicited much stronger STING activation than TfR/PDL1‐GBS and cGAMP treatments (Figure 4d and Figures S16 and S17), suggesting that effective activation of cGAS‐STING requires the release of short GBS. In addition, the transcription levels of IFN‐β and CXCL10 (a crucial chemokine involved in the cGAS‐STING signaling pathway and T‐cell recruitment) in TfR/PDL1‐GBS@ZnO‐treated cells increased dramatically as quantified by qPCR (Figure 4e,f) [13].

We next evaluated whether TfR/PDL1‐GBS@ZnO induced cGAS‐STING activation promotes bone marrow–derived Dendritic Cells (BMDCs) maturation. In a transwell co‐culture system, TfR/PDL1‐GBS@ZnO‐treated 4T1 cells were seeded in the lower chamber and murine BMDCs in the upper chamber, allowing cytokine exchange without cell contact (Figure 4g). After 48 h, flow cytometry revealed that 35.4% of BMDCs expressed the maturation markers CD80/CD86, and TfR/PDL1‐RS@ZnO induced only 14.0% BMDCs maturation, indicating that GBS is essential for robust pathway activation and that the DNA nanostructure itself contributes minimal nonspecific stimulation (Figure 4h,i and Figure S13). In addition, to further demonstrate the clinical relevance of STTAC, we evaluated the ability of TfR/PDL1‐GBS@ZnO to induce the maturation of human dendritic cells (Figure S18). Together, these results demonstrate that TfR/PDL1‐GBS@ZnO strongly activates the cGAS‐STING axis in tumor cells, leading to IFN‐β secretion and enhanced BMDCs maturation, thereby establishing a foundation for in vivo therapeutic applications.

### 2.5 TfR/PDL1‐GBS@ZnO–Mediated Immunogenic Cell Death (ICD) and Immune Activation

**FIGURE 5:**
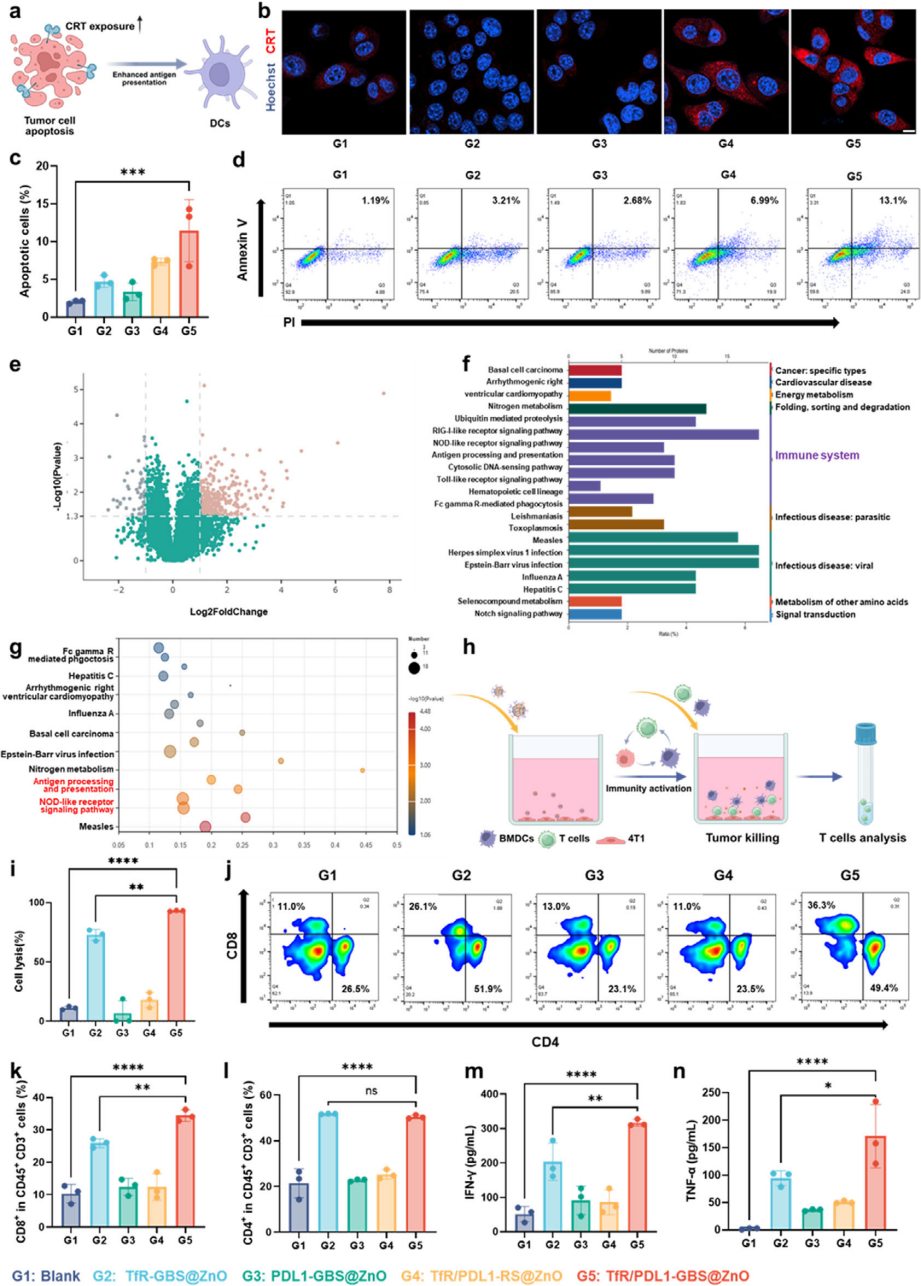
TfR/PDL1‐GBS@ZnO–mediated immunogenic cell death and immune activation. (a) Schematic of TfR/PDL1‐GBS@ZnO ‐induced immunogenic cell death (ICD) in tumor cells and enhanced DCs antigen presentation, Created in BioRender.com. (b) Immunofluorescence analysis of CRT expression in 4T1 cells after different treatments. Scale bar = 10 µm. Flow cytometric analysis (d) and statistical quantification (c) of apoptosis in 4T1 cells after different treatments. (e) Volcano plot showing differentially expressed proteins between PBS and TfR/PDL1‐GBS@ZnO treated 4T1cells, *p* < 0.05. (f) KEGG pathway enrichment of differentially expressed proteins between PBS and TfR/PDL1‐GBS@ZnO groups, displayed as a category‐annotated bar chart ranked by enrichment fold. (g) KEGG pathway enrichment of differentially expressed proteins between PBS and TfR/PDL1‐GBS@ZnO groups, visualized as a dot plot integrating enrichment factor (x‐axis), hit counts (bubble size), and significance (color, −log10 *p* value). h) T cell activation and tumor cell killing in a tumor cell–immune cell co‐culture system, Created in BioRender.com. (i) Evaluation of T cell–mediated killing of 4T1 cells. Flow cytometry analysis (j) and statistical quantification of CD8^+^ (k) and CD4^+^ (l) T cells within the immune cell population. IFN‐γ (m) and TNF‐α (n) secretion after different treatments. Data are presented as mean ± S.D., *n* = 3, Significance (one‐way ANOVA) is labeled above the data, ^*^ *p* < 0.05, ^**^ *p* < 0.01, ^***^ *p* < 0.001, ^****^ *p* < 0.0001.

Previous studies have shown that PD‐L1 degradation can induce immunogenic cell death (ICD) in tumor cells [35], leading to the membrane exposure of the ICD‐related biomarker calreticulin (CRT) that promotes Dendritic Cells (DCs) recognition and immune responses (Figure 5a). We first examined cell viability after STTAC treatments and found TfR/PDL1‐RS@ZnO and TfR/PDL1‐GBS@ZnO induced potent tumor cell death, with TfR/PDL1‐GBS@ZnO exerting a stronger cell‐killing effect (Figure S19). Then, we assessed CRT expression in tumor cells by immunofluorescence, low CRT expression was detected of 4T1 cells treated with TfR‐GBS@ZnO and PDL1‐GBS@ZnO, whereas TfR/PDL1‐RS@ZnO and TfR/PDL1‐GBS@ZnO groups exhibited elevated CRT expression. These findings indicate that CRT expression is primarily dependent on PD‐L1 degradation (Figure 5b). Furthermore, Annexin V‐FITC/propidium iodide (PI) staining revealed that TfR/PDL1‐GBS@ZnO primarily induces programmed apoptosis (Figure 5c,d).

Next, we performed proteomic profiling analysis of 4T1 cells treated with PBS and TfR/PDL1‐GBS@ZnO.

Overall, the proteomic changes were relatively selective, with only a small fraction of proteins showing significant differential expression (Figure 5e). Kyoto Encyclopedia of Genes and Genomes (KEGG) pathway analysis revealed the top 20 enriched pathways among the differentially expressed proteins. TfR/PDL1‐GBS@ZnO treatment enhanced multiple immune‐related pathways, including the NOD‐like receptor signaling pathway, RIG‐I‐like receptor signaling pathway, and cytosolic DNA‐sensing pathway (Figure 5f,g) [36, 37]. Notably, antigen processing and presentation were also markedly enriched, indicating enhanced tumor immunogenicity and improved susceptibility to T‐cell recognition, consistent with our previous results (Figure 5g,b).

Furthermore, Gene Set Enrichment Analysis (GSEA) revealed significant enrichment of autophagy‐ and endocytosis‐related pathways, all of which were upregulated upon TfR/PDL1‐GBS@ZnO treatment. These findings indicated that TfR/PDL1‐GBS@ZnO activated the STING pathway, which further enhanced lysosomal degradative capacity through the upregulation of endocytosis and autophagy (Figure S20).

We further performed tumor–immune cell co‐culture experiments to mimic the stimulation of the tumor immune microenvironment by TfR/PDL1‐GBS@ZnO (Figure 5h). This in vitro model does not fully recapitulate the complexity of the in vivo immune microenvironment, where dendritic cell maturation and subsequent T cell activation occur in a temporally coordinated manner. Therefore, this experiment serves as a simplified model to evaluate T cell activation and tumor cell killing. Upon TfR/PDL1‐GBS@ZnO stimulation, BMDCs underwent maturation and presented tumor antigens to T cells, leading to T‐cell activation, expansion, and tumor cell killing. TfR/PDL1‐GBS@ZnO treatment resulted in a marked increase in the proportion of CD4^+^ and CD8^+^ T cells within the immune cell population (Figure 5j–l), accompanied by a strong tumor cell killing efficiency (Figure 5i), indicating robust T‐cell activation and cytotoxicity. Notably, TfR‐GBS@ZnO also achieved substantial tumor cell killing. However, its inability to degrade tumor cell PD‐L1 compromises T‐cell–mediated cytotoxicity to some extent. Synergistic cGAS‐STING activation and PD‐L1 downregulation exerted enhanced tumor cell killing effect. Elevated secretion of IFN‐γ and TNF‐α provides further evidence of dendritic cell maturation and T‐cell activation (Figure 5m,n). To further evaluate the clinical relevance of STTAC, we established a patient‐derived tumor‐like cell cluster (PTC) model from clinical lung adenocarcinoma samples (Figure S21) [38]. 3D imaging revealed that TfR/PDL1‐GBS@ZnO effectively degraded PD‐L1 in the PTC model. Collectively, these results demonstrate that the synergistic effects of TfR/PDL1‐GBS@ZnO‐mediated PD‐L1 degradation and STING activation contribute to remodeling the tumor immune microenvironment.

### 2.6 TfR/PDL1‐GBS@ZnO‐Mediated Immune Cytotoxicity Against Tumors In Vivo

We established a 4T1 tumor‐bearing Balb/c mouse model to evaluate the in vivo biodistribution of TfR/PDL1‐GBS@ZnO. 24 h after injection of Cy5‐labeled TfR/PDL1‐GBS@ZnO (2 mg/kg), major organs and tumors were harvested, more intensified fluorescence in tumors was observed from TfR/PDL1‐GBS@ZnO‐treated mice, indicating that TfR and PD‐L1 bispecific tumor recognition are critical for active tumor targeting (Figure S22).

**FIGURE 6:**
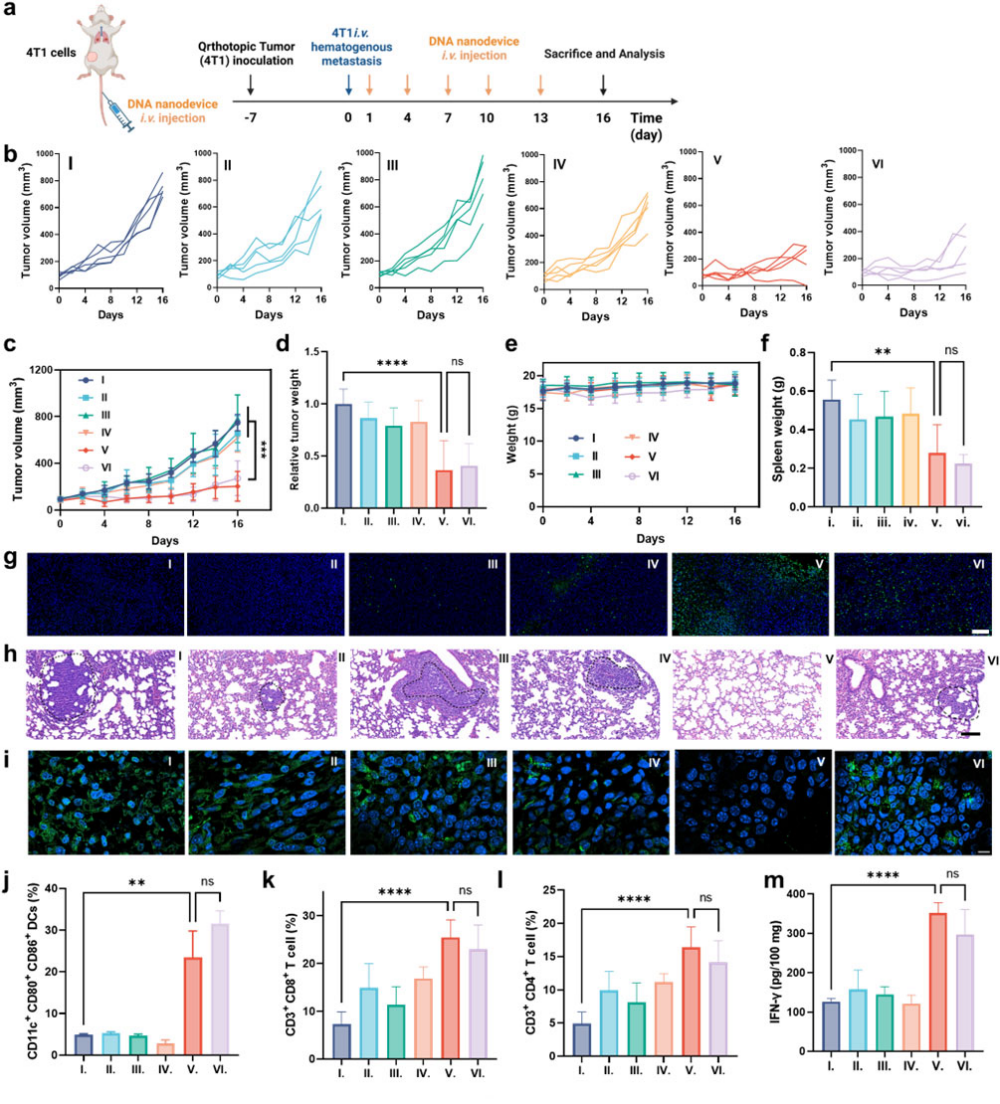
TfR/PDL1‐GBS@ZnO for the effective immunotherapy of primary tumors and inhibited lung metastasis. (a) Schematic diagram of the timeline for evaluating the immune activation of TfR/PDL1‐GBS@ZnO in a 4T1 tumor model, Created in BioRender.com. (i) Blank, (ii) TfR‐GBS@ZnO, (iii) PDL1‐GBS@ZnO, (iv) TfR/PDL1‐RS@ZnO, (v) TfR/PDL1‐GBS@ZnO, (vi) diABZI. (b) Individual tumor growth curves of 4T1 tumor‐bearing mice after different treatment, *n* = 5. (c) Tumor growth curves and relative tumor weight (d) of 4T1 tumor‐bearing mice after different treatment, *n* = 5. (e) Mice body weight of different treatment groups, *n* = 5. (f) Spleen weight of 4T1 tumor‐bearing mice after different treatment, *n* = 5. (g) TUNEL analysis of tumor sections. Scale bar = 50 µm. (h) H&E analysis of lung with metastatic 4T1 tumors. Scale bar = 100 µm. (i) Immunofluorescence analysis of PD‐L1 degradation in tumor section. Scale bar = 20 µm. (j) Flow cytometric analysis of CD11c^+^ CD80^+^ CD86^+^ cell percentage in TDLNs, *n* = 3. Flow cytometry analysis of the corresponding CD8^+^ T cell (k) and CD4^+^ T cell (l) percentage in tumor tissues, *n* = 5. (m) Elisa analysis of the secretion levels of IFN‐γ in tumor tissue, *n* = 5. Data are presented as mean ± S.D., Significance (one‐way ANOVA) is labeled above the data, ^**^ *p* < 0.01, ^***^ *p* < 0.001, ^****^ *p* < 0.0001.

To evaluate the therapeutic efficacy of TfR/PDL1‐GBS@ZnO in vivo, we established an orthotopic and lung metastatic 4T1 models in Balb/c mice (Figure 6a). When tumors reached 100 mm^3^, mice were randomized into six treatment groups and injected every three days. diABZI (2 mg/kg) [4] served as a benchmark control. After 16 days, TfR/PDL1‐GBS@ZnO and diABZI treatment led to significant tumor growth suppression, whereas TfR‐GBS@ZnO, PDL1‐GBS@ZnO or TfR/PDL1‐RS@ZnO exhibited poor effectiveness (Figure 6b–d and Figure S23). And no significant body‐weight changes were observed, indicating favorable tolerability (Figure 6e).

Next, we examined the spleen, a key immune organ, to assess systemic antitumor immune responses. As shown in Figure 6f, spleen weights were higher in the Blank, TfR‐GBS@ZnO, PDL1‐GBS@ZnO, and TfR/PDL1‐RS@ZnO groups, which may reflect splenomegaly attributable to tumor progression–associated extramedullary hematopoiesis and the accumulation of erythroid progenitors [39]. In contrast, mice treated with TfR/PDL1‐GBS@ZnO or diABZI exhibited lower spleen weights (Figure 6f). Histological analyses by TUNEL and H&E staining revealed extensive apoptosis in tumors from TfR/PDL1‐GBS@ZnO and diABZI‐treated groups (Figure 6g and Figure S24). In metastatic assays, intravenous injection of 4T1 cells resulted in severe pulmonary metastases in PBS and control nanodevice groups, modest nodules in diABZI‐treated mice, but almost complete abrogation of lung metastases with TfR/PDL1‐GBS@ZnO (Figure 6h and Figure S25).

Mechanistically, immunofluorescence confirmed robust PD‐L1 degradation in tumors treated with TfR/PDL1‐GBS@ZnO, but not in groups lacking TfR or PD‐L1 aptamers (Figure 6i), highlighting the necessity of aptamer‐mediated targeting. Flow cytometry further demonstrated TfR/PDL1‐GBS@ZnO‐induced maturation of 23% of DCs in tumor‐draining lymph nodes, comparable to diABZI (Figure 6j). Importantly, TfR/PDL1‐GBS@ZnO significantly enhanced intratumoral infiltration of CD8^+^ T cells, elevated IFN‐γ secretion, and improved CD8^+^ cytotoxic responses (Figure 6k–m and Figure S26). In addition, the in vivo safety of the STTAC system was evaluated. TfR/PDL1‐GBS@ZnO was nearly undetectable in the bloodstream within 12 h post‐administration (Figure S27). The serum levels of aspartate aminotransferase (AST), alanine aminotransferase (ALT), and blood urea nitrogen (BUN) were monitored over time and showed no significant differences compared with those of healthy mice, indicating minimal systemic toxicity (Figure S28). Together, these findings demonstrate that TfR/PDL1‐GBS@ZnO not only effectively degrade tumor PD‐L1 in vivo but also activate endogenous intratumoral STING signaling, thereby remodeling the immunosuppressive TNBC microenvironment and achieving potent antitumor efficacy against both primary and metastatic 4T1 tumors.

## 3 Conclusion

In summary, we have constructed a DNA nanodevice (TfR/PDL1‐GBS@ZnO) that integrates lysosome‐targeted degradation of PD‐L1 with activation of the cGAS‐STING pathway. This platform addresses key limitations of current immunotherapies by enabling tumor‐specific, dual‐action immune modulation within a single, nucleic acid‐based structure. The nanodevice hijacks the TfR for efficient lysosomal delivery, where acid‐triggered release of both PD‐L1‐targeting aptamers and STING‐activating dsDNA fragments occurs. We demonstrate a synergistic crosstalk wherein STING activation further amplifies lysosomal function and PD‐L1 degradation, leading to potent reprogramming of the tumor microenvironment. This is evidenced by enhanced dendritic cell maturation, robust CD8^+^ T cell infiltration, and significant suppression of both primary tumor growth and metastasis in aggressive triple‐negative breast cancer models. By marrying the principles of targeted protein degradation with innate immune activation, our work establishes a versatile and chemically programmable framework for next‐generation cancer immunotherapy. Future efforts will focus on optimizing the uniformity and scalability of the nanodevice to accelerate its translational potential.

## 4 Experimental Section

### 4.1 Preparation of TfR/PDL1‐GBS@Zno

As an example, circle template‐PDL1‐GBS or circle template‐TfR‐GBS were prepared by mixing 20 µL of the primer (10 µm), 20 µL of template (10 µm), and 48.5 µL ddH_2_O. The mixture was first heated at 95°C for 5 min and slowly cooled to room temperature. Then, 10 µL of 10×T4 DNA ligase reaction buffer (400 mm Tris‐HCl, 100 mm MgCl_2_, 100 mm DTT, 5 mm ATP) and 2.5 µL of T4 DNA ligase (1000 U/µL) were added to the mixture. The mixture was incubated at 16°C overnight and then heated at 65°C for 10 min to terminate the reaction. RCA reaction was performed in a volume of 100 µL containing 25 µL circle template‐PDL1‐GBS and circle template‐TfR‐GBS (2 µm), 10 µL of 10 × phi29 DNA polymerase reaction buffer (330 mm Tris‐acetate, 100 mm Mg‐acetate, 660 mm K‐acetate, 1% (v/v) Tween 20, 10 mm DTT), 8 µL dNTPs (25 mm), 10 µL phi29 DNA polymerase (10 U/µL) and 47 µL of ddH_2_O. RCA reaction was performed at 37°C for 2 h and inactivated by heating at 65°C for 10 min. As before paper reported [30], TfR/PDL1‐GBS@ZnO were synthesized by adding extra 10 mm ZnO nanoparticles in the RCA mixture. And the resultant products were washed with ultrapure water and centrifuged at 12000 rpm for 15 min three times and then were sonicated and pipetted several times to redisperse the nanodevice. Then TfR/PDL1‐GBS@ZnO was saved at 4°C and sonicated briefly before each use to ensure proper nanoparticle dispersion.

### 4.2 Confocal Laser Scanning Microscopy (CLSM) Assay

For fluorescence imaging of TfR/PDL1‐GBS@ZnO endocytosis by cells, Cy3‐labeled dCTP (dCTP–Cy3) was co‐incubated in the RCA reaction to introduce a fluorescent tag into TfR/PDL1‐GBS@ZnO. 4T1 cells were incubated with different groups in 1 mL DMEM medium for 3 h at 37°C, following washed three times with PBS to remove free TfR/PDL1‐GBS@ZnO. Then washed three times with PBS. And cell imaging was performed under CLSM (Carl Zeiss GmbH, LSM980, Jena, Germany).

For Immuno Fluorescence, 4T1 cells were incubated in a confocal dish. And cells were incubated with TfR/PDL1‐GBS@ZnO (20 µg/mL) in 1 mL DMEM medium for 12 h at 37°C. The cells were first washed three times with PBS. Cells were fixed with 4% paraformaldehyde and washed three times with PBS, then blocked with 200 µL of 5% BSA/TBST at room temperature for 1 h. Next, apply 100 µL primary antibody (prepared with 1% BSA/TBST) to the dishes overnight at 4°C. The next day, cells were washed at least 3 times with PBS. After incubating the secondary antibody (prepared with 1% BSA/TBST) at room temperature for 3 h, the cells used Hoechst (1% BSA/TBST dilution) to stain cell nuclei at room temperature for 10 min. Wash with PBS at least 3 times, 5 min each time. The cells were ready for IF imaging with the CLSM.

### 4.3 Western Blot (WB)

Cells were seeded in 12‐well plate (Biofil). When the cells reached 80% confluence, the medium was changed and incubated with 20 µg/mL TfR/PDL1‐GBS@ZnO for different hours in the incubator. Then the dishes were put on the ice to stop the stimulation and washed three times by precooling PBS, then lysed with lysis buffer (RIPA buffer with 1% phosphatase inhibitors and protease inhibitor). The cell lysates were centrifuged at 15000 rpm for 20 min, then retain the supernatant, saved in the −20°C before use. Then the protein samples (30 µg total protein) were incubated with 5× loading sample buffer at 95°C for 10 min. The samples were then analyzed by 12% sodium dodecyl sulfate polyacrylamide gel electrophoresis (SDS‐PAGE) and transferred to nitrocellulose filter membranes at 300 mA for 120 min. After blocking in TBST with 5% BSA for 3 h, the membranes were incubated overnight at 4°C with primary antibodies. After washing 3 times, the membranes were incubated with appropriate secondary antibodies for 2 h at room temperature. The membranes were imaged by a MiniChemi Imaging System (SINSAGE, Beijing, China) using chemiluminescence.

### 4.4 STING Pathway Activation and BMDCs Co‐Culture

Bone marrow‐derived dendritic cells (BMDCs) were isolated from femurs of BALB/c mice and cultured in RPMI‐1640 supplemented with 10% FBS, 20 ng/mL GM‐CSF, and 10 ng/mL IL‐4 (PeproTech). 4T1 cells were seeded in the lower chamber of Transwell inserts at a density of 5 × 10^4^ cells/well. BMDCs were seeded in the upper chamber. TfR/PDL1‐GBS@ZnO (20 µg/mL) were added into lower chamber. After 48 h of co‐culture, BMDCs were harvested for flow cytometric analysis. BMDCs maturation was assessed by staining with anti CD11c, anti‐CD80 and anti‐CD86 antibodies.

For human DCs maturation, MDA‐MB‐231 cells were seeded in the lower chamber of Transwell inserts at a density of 5 × 10^4^ cells/well. Human DCs were seeded in the upper chamber. Subsequent experiments were performed as described above.

### 4.5 In Vivo Therapeutic Experiments

All experimental protocols were approved by the Committee on the Ethics of Animal Experiments of School of Biomedical Sciences Hunan University [HNU‐IACUC‐2022‐121]. Balb/c mice were purchased from Gempharmatech Co., Ltd (Chengdu, China). Female balb/c mice, 4–6 weeks of age, For TfR/PDL1‐GBS@ZnO reprograme tumor immunity in vivo, mice were subcutaneously implanted with 4T1 cells (5 × 10^5^ per mouse) to construct tumor‐bearing mouse model. And for lung metastatic model, additional 4T1 cells (5 × 10^5^) were intravenous injected in mice. When tumor volume reached about 50–100 mm^3^, mice were randomly divided into four groups and given different treatments. TfR/PDL1‐GBS@ZnO was administered via intravenous injection (2 mg/kg) at 3‐day intervals. The tumor volume measured by vernier caliper and mice weight were recorded every two days. The tumor volume was calculated according to the formula V = (L × W^2^)/2 (L: longer diameter of tumor, W: shorter diameter of tumor). On day 16, the mice were sacrificed for tumor isolation and experimental analysis.

## Author Contributions

**Haoxiang Li**: conceptualization, methodology, data curation, validation, formal analysis, writing – original draft. **Jianjun He**: writing – review and editing, conceptualization, methodology, supervision, funding acquisition, investigation, formal analysis. **Shasha Sun**: methodology, data curation, validation. **Jun Cao**: funding acquisition, writing – review and editing, supervision, project administration. **Jian‐Hui Jiang**: funding acquisition, writing – review and editing, supervision, resources, project administration. **Min Hou**: methodology, data curation, investigation, formal analysis.

## Conflicts of Interest

The authors declare no conflicts of interest.

## Supporting information




**Supporting File**: advs75855‐sup‐0001‐SuppMat.docx.

## Data Availability

The data that support the findings of this study are available from the corresponding author upon reasonable request.

## References

[1] J. Sun , J. Wu , F. Du , X. Chen , and Z. J. Chen , “Cyclic GMP‐AMP Synthase Is a Cytosolic DNA Sensor That Activates the Type I Interferon Pathway,” Science 1 (2013): 786–791. 10.1126/science.1232458 PMC3863629 23258413

[2] X. Li , M. Wenes , P. Romero , S. C. Huang , S. M. Fendt , and P. C. Ho , “Navigating Metabolic Pathways to Enhance Antitumour Immunity and Immunotherapy,” Nature Reviews Clinical Oncology 16 (2019): 425–441, 10.1038/s41571-019-0203-7. 30914826

[3] J. J. Havel , D. Chowell , and T. A. Chan , “The Evolving Landscape of Biomarkers for Checkpoint Inhibitor Immunotherapy,” Nature Reviews Cancer 19 (2019): 133–150, 10.1038/s41568-019-0116-x. 30755690 PMC6705396

[4] J. M. Ramanjulu , G. S. Pesiridis , J. Yang , et al., “Design of Amidobenzimidazole STING Receptor Agonists with Systemic Activity,” Nature 564 (2018): 439–443. 30405246 10.1038/s41586-018-0705-y

[5] B. S. Pan , S. A. Perera , J. A. Piesvaux , et al., “An Orally Available Non‐nucleotide STING Agonist with Antitumor Activity,” Science 369 (2020): aba6098. 10.1126/science.aba6098 32820094

[6] H. Song , L. Chen , X. Pan , et al., “Targeting Tumor Monocyte‐Intrinsic PD‐L1 by Rewiring STING Signaling and Enhancing STING Agonist Therapy,” Cancer Cell 43 (2025): 503–518.e10, 10.1016/j.ccell.2025.02.014. 40068600

[7] Y. Liu , W. N. Crowe , L. Wang , et al., “An Inhalable Nanoparticulate STING Agonist Synergizes with Radiotherapy to Confer Long‐term Control of Lung Metastases,” Nature Communications 10 (2019): 5108, 10.1038/s41467-019-13094-5. PMC6841721 31704921

[8] Y. Fan , Y. Gao , L. Nie , et al., “Targeting LYPLAL1‐mediated cGAS Depalmitoylation Enhances the Response to Anti‐Tumor Immunotherapy,” Molecular Cell 83 (2023): 3520–3532.e7, 10.1016/j.molcel.2023.09.007. 37802025

[9] P. Sharma and J. P. Allison , “The Future of Immune Checkpoint Therapy,” Science 348 (2015): 56–61. 25838373 10.1126/science.aaa8172

[10] D. B. K. Juan Fu , M. Leong , L. H. Glickman , et al., “An Inflammation‐Targeting Hydrogel for Local Drug Delivery in Inflammatory Bowel Disease,” Science Translational Medicine 7 (2015): 1–11. 10.1126/scitranslmed.aaa5657 PMC4825054 26268315

[11] Y. Hai , Z. Xun , L. Yang , Z. Cai , Z. Wu , and J. H. Jiang , “Chemically Inducible Cyclic Dinucleotides as Self‐Deliverable STING Agonists with Enhanced Antitumor Immunity,” Journal of the American Chemical Society 147 (2025): 28429–28441, 10.1021/jacs.5c09626. 40701545

[12] J. Gan , J. Lei , Y. Li , M. Lu , X. Yu , and G. Yu , “Manganese Oxide‐Incorporated Hybrid Lipid Nanoparticles Amplify the Potency of mRNA Vaccine via Oxygen Generation and STING Activation,” Journal of the American Chemical Society 146 (2024): 32689–32700, 10.1021/jacs.4c12166. 39552027

[13] Q.‐R. Li , X. Zhang , C. Zhang , et al., “Biomineralized Engineered Bacterial Outer Membrane Vesicles as cGAS‐STING Nanoagonists Synergize with Lactate Metabolism Modulation to Potentiate Immunotherapy,” Journal of the American Chemical Society 147 (2025): 24555–24572, 10.1021/jacs.5c05148. 40601938

[14] T. Yang , D. Huang , C. Li , et al., “Rolling Microneedle Electrode Array (RoMEA) Empowered Nucleic Acid Delivery and Cancer Immunotherapy,” Nano Today 36 (2021): 101017.

[15] T. Chen , Q. Li , Z. Liu , Y. Chen , F. Feng , and H. Sun , “Peptide‐based and Small Synthetic Molecule Inhibitors on PD‐1/PD‐L1 Pathway: A New Choice for Immunotherapy?,” European Journal of Medicinal Chemistry 161 (2019): 378–398, 10.1016/j.ejmech.2018.10.044. 30384043

[16] J. Zhao , A. Tong , J. Liu , M. Xu , and P. Mi , “Tumor‐targeting Nanocarriers Amplified Immunotherapy of Cold Tumors by STING Activation and Inhibiting Immune Evasion,” Science Advances 11 (2025): adr1728. 10.1126/sciadv.adr1728 PMC12204131 40577452

[17] W. Su , M. Tan , Z. Wang , et al., “Targeted Degradation of PD‐L1 and Activation of the STING Pathway by Bone‐Targeted Nanoparticles for Enhanced Cancer Immunotherapy,” Angewandte Chemie International Edition 62 (2023): 202218128. 10.1002/anie.202218128 36647763

[18] X. Liu , Z. Zhao , X. Xu , et al., “Mobilizing STING Pathway via a Cationic Liposome to Enhance Doxorubicin‐Induced Antitumor Immunity,” Advanced Functional Materials 35 (2024): 2416406.

[19] Z. Chen , Z. Feng , S. Wang , and J. Zhang , “Recent Progress in Solid‐State Lithium Batteries through Cathode Microstructure Engineering,” Advanced Science 12 (2025): 13455. 10.1002/advs.202513455 PMC12752667 41255235

[20] H. Yuan , C. Qiu , X. Wang , et al., “Engineering Semiconducting Polymeric Nanoagonists Potentiate cGAS‐STING Pathway Activation and Elicit Long Term Memory against Recurrence in Breast Cancer,” Advanced Materials 37 (2025): 2406662, 10.1002/adma.202406662. 39629527

[21] C. Qiu , F. Xia , Q. Tu , et al., “Multimodal Lung Cancer Theranostics via Manganese Phosphate/Quercetin Particle,” Molecular Cancer 24 (2025): 43. 39905491 10.1186/s12943-025-02242-9 PMC11796208

[22] F. Xia , Y. Lu , Z. Gong , et al., “Cancer Immunotherapy Based on the Bidirectional Reprogramming of the Tumor Microenvironment by a “Brakes off/ Step on the Accelerator” Core‐Shell Manganese Phosphate/siPD‐L1 Modulator,” Exploration 5 (2025): 270009. 40585771 10.1002/EXP.70009 PMC12199384

[23] T. Guan , Z. Chen , X. Wang , et al., “Harnessing Mn^2+^ Ions and Antitumor Peptides: A Robust Hydrogel for Enhanced Tumor Immunotherapy,” Journal of the American Chemical Society 147 (2025): 6523–6535, 10.1021/jacs.4c14700. 39950218

[24] X. Li , F. Yu , and L. Li , “Tandem‐Controlled Dynamic DNA Assembly Enables Temporally‐Selective Orthogonal Regulation of cGAS–STING Stimulation,” Angewandte Chemie International Edition 64 (2024): 202417916, 10.1002/anie.202417916. 39526866

[25] K. Morihiro , H. Osumi , S. Morita , et al., “Oncolytic Hairpin DNA Pair: Selective Cytotoxic Inducer through MicroRNA‐Triggered DNA Self‐Assembly,” Journal of the American Chemical Society 145 (2022): 135–142, 10.1021/jacs.2c08974. 36538570

[26] K. M. Mayle , A. M. Le , and D. T. Kamei , “The Intracellular Trafficking Pathway of Transferrin,” Biochimica et Biophysica Acta (BBA)—General Subjects 1820 (2012): 264–281, 10.1016/j.bbagen.2011.09.009. 21968002 PMC3288267

[27] D. Zhang , J. Duque‐Jimenez , F. Facchinetti , et al., “Transferrin Receptor Targeting Chimeras for Membrane Protein Degradation,” Nature 638 (2024): 787–795, 10.1038/s41586-024-07947-3. 39322661 PMC11839386

[28] Z. R. Crook , G. P. Sevilla , P. Young , et al., “CYpHER: Catalytic Extracellular Targeted Protein Degradation with High Potency and Durable Effect,” Nature Communications 15 (2024): 8731. 10.1038/s41467-024-52975-2 PMC11464628 39384759

[29] A.‐M. Herzner , C. A. Hagmann , M. Goldeck , et al., “Sequence‐specific Activation of the DNA Sensor cGAS by Y‐form DNA Structures as Found in Primary HIV‐1 cDNA,” Nature Immunology 16 (2015): 1025–1033, 10.1038/ni.3267. 26343537 PMC4669199

[30] J. Wang , H. Wang , H. Wang , et al., “Nonviolent Self‐catabolic DNAzyme Nanosponges for Smart Anticancer Drug Delivery,” ACS Nano 13 (2019): 5852–5863. 31042356 10.1021/acsnano.9b01589

[31] Y. Xu , Q. Wang , J. Wang , et al., “The cGAS‐STING Pathway Activates Transcription Factor TFEB to Stimulate Lysosome Biogenesis and Pathogen Clearance,” Immunity 58 (2025): 309–325.e6, 10.1016/j.immuni.2024.11.017. 39689715

[32] M. A. Suter , N. Y. Tan , C. H. Thiam , et al., “cGAS–STING Cytosolic DNA Sensing Pathway Is Suppressed by JAK2‐STAT3 in Tumor Cells,” Scientific Reports 11 (2021): 7243, 10.1038/s41598-021-86644-x. 33790360 PMC8012641

[33] Y. Miao , Q. Gao , M. Mao , et al., “Bispecific Aptamer Chimeras Enable Targeted Protein Degradation on Cell Membranes,” Angewandte Chemie International Edition 60 (2021): 11267–11271, 10.1002/anie.202102170. 33634555

[34] B. Zhang , R. K. Brahma , L. Zhu , et al., “Insulin‐Like Growth Factor 2 (IGF2)‐Fused Lysosomal Targeting Chimeras for Degradation of Extracellular and Membrane Proteins,” Journal of the American Chemical Society 145, no. 44 (2023): 24272–24283, 10.1021/jacs.3c08886. 37899626

[35] Y. Li , X. Liu , L. Yu , et al., “Covalent LYTAC Enabled by DNA Aptamers for Immune Checkpoint Degradation Therapy,” Journal of the American Chemical Society 145 (2023): 24506–24521. 10.1021/jacs.3c03899 37910771

[36] L. Chen , S. Cao , Z. Lin , S. He , and J. Zuo , “NOD‐like Receptors in Autoimmune Diseases,” Acta Pharmacologica Sinica 42, no. 11 (2021): 1742–1756. 33589796 10.1038/s41401-020-00603-2 PMC8564530

[37] K. Onomoto , K. Onoguchi , and M. Yoneyama , “Regulation of RIG‐I‐Like Receptor‐Mediated Signaling: Interaction between Host and Viral Factors,” Cellular & Molecular Immunology 18, no. 3 (2021): 539–555, 10.1038/s41423-020-00602-7. 33462384 PMC7812568

[38] Y. Hou , H. L. Liang , X. Yu , et al., “Radiotherapy and Immunotherapy Converge on Elimination of Tumor‐Promoting Erythroid Progenitor Cells through Adaptive Immunity,” Science Translational Medicine 13 (2021): abb0130. 10.1126/scitranslmed.abb0130 PMC8710940 33627484

[39] Y. Hou , H. Liang , X. Yu , et al., “Radiotherapy and Immunotherapy Converge on Elimination of Tumor‐promoting Erythroid Progenitor Cells through Adaptive Immunity,” Science Translational Medicine 13 (2021): abb0130, 10.1126/scitranslmed.abb0130. PMC8710940 33627484

