# Supplementary material for: Synergistic Remodeling of Tumor Immune Microenvironment via a DNA Nanodevice Integrating STING Activation and Lysosome‐Targeted PD‐L1 Degradation
Source: Adv Sci (Weinh). 2026 May 27:e75855. Online ahead of print. doi: 10.1002/advs.75855 (PMC13336052; doi:10.1002/advs.75855)
Supplement: Supplementary file 1 — Supporting File: advs75855‐sup‐0001‐SuppMat.docx. [file ADVS-9999-e75855-s001.docx]

Supporting Information

**Synergistic Remodeling of Tumor Immune Microenvironment via a DNA Nanodevice Integrating STING Activation and Lysosome-Targeted PD-L1 Degradation**

Haoxiang Li^1^, Min Hou^2, *^, Shasha Sun^1^, Jun Cao^1^, Jian-Hui Jiang^1^, Jianjun He^1, 3*^

^1^School of Biomedical Sciences, State Key Laboratory of Chemo/Bio-Sensing and Chemometrics, College of Chemistry and Chemical Engineering, Hunan University, Changsha 410082, China.

^2^School of Physics and Chemistry, Hunan First Normal University, Changsha, 410205, China.

^3^FuRong Laboratory, Changsha, 410078, China.

E-mail: caojun10@126.com, jianjunh@hnu.edu.cn

**
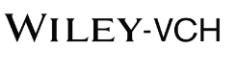
SUPPORTING INFORMATION**


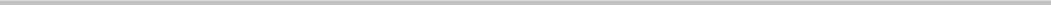


**Materials and Methods**

**Materials.** All DNA oligonucleotides and primers presented in Table S1 were synthesized and HPLC purified by General Biological Engineering Technology & Co. Ltd. (Anhui, China). T4 ligase and Phi 29 polymerase were purchased from Sangon Biotech Co. Ltd. (Shanghai, China). TFEB anitbody, p-TFEB antibody and Mouse-Reactive STING Pathway Antibody Sampler Kit (16029) were purchased from CST signaling Technology, Inc. (Boston, USA). Cell Counting Kit-8 was obtained from APExBIO (Houston, American). Annexin V-FITC/PI Apoptosis Detection kit were purchased from Abbkine (Hubei, China). CD3 Monoclonal Antibody (14-0037-82) were purchased from Thermo Fisher Scientific (Waltham, USA). PD-L1/CD274 Monoclonal antibody were purchased from proteintech (Hubei, China). FITC anti-mouse CD80 Antibody, PE anti-mouose CD11c Antibody, APC anti-mouse CD86 Antibody, FITC anti-human CD80 Antibody, APC anti-human CD86 Antibody, PE anti-human CD11c Antibody, FITC anti-mouse CD3 Antibody, PE anti-mouse CD4 Antibody and APC anti-mouse CD8 Antibody were purchased from Biolegend (California, USA). p-62 antibody, LC3B antibody, Alexa Fluor 647-labeled Goat Anti-Mouse IgG (A0473) and Alexa Fluor 488-labeled Goat Anti-Rabbit IgG (A0423) were purchased from Beyotime (Shanghai, China). Dulbecco's Modified Eagle medium (DMEM), RPMI 1640 medium (1640) and 1×Phosphate Buffered Saline (PBS) were purchased from Servicebio (Hubei, China). Fetal bovine serum (FBS) were purchased from CellMax (Beijing, China). IFN-β ELISA kit were purchased from Biolegend Bioscience Inc (San Diego, USA). AST and ALT ELISA kit were purchased from ZCI bio (Shanghai, China). Urea (BUN) colorimetric kit was purchased from ZCI bio (Shanghai, China). IFN-γ (abs510007) and TNF-α (abs510006) ELISA kit were purchased from Absin Bioscience Inc (Shanghai, China).

**Cell culture.** 4T1 cells, Hela cells, A549 cells, MDA-MB-231 cells were obtained from the Procell. Cells were maintained in DMEM medium supplemented with 10% FBS, 1% penicillin-streptomycinat 37 °C in 5% CO_2_. Dendritic cells (DCs) cells were maintained in RPMI 1640 medium supplemented with 10% FBS, 1% penicillin-streptomycin, 1% GultaMax and 20 ng/mL GM-CSF 37 °C in 5% CO_2_. For immune cell co-culture assays, DCs and T cells were isolated from BALB/c mice. human DCs were purchased from SCHBio (Shanghai, China).

**Gel electrophoresis.** All DNA samples (10 μL) were mixed with 6× loading buffer (2 μL). TBE buffer was used as the running buffer. For agarose gel electrophoresis, 1% agarose gel was used, electrophoresis was carried out in 1× TBE at 120 V for 40 min. Subsequently, the gel was analyzed using ChampGel ® 5000 Gel Imaging System (SINSAGE, Beijing, China).

**
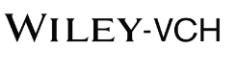
SUPPORTING INFORMATION**


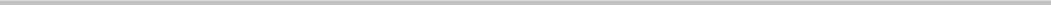


**Dynamic Light Scattering (DLS) and Zeta Potential.** Hydrodynamic size and surface zeta potential were measured using a Malvern Zetasizer Nano ZS. Samples were dispersed in PBS at a concentration of 0.1 mg/mL.

**Scanning electron microscope (SEM) assay.** The silicon wafers were sequentially washed with ultrapure water, acetone, and ethanol, and then dried. 20 µL of the DNA samples were dropped onto the surface of silicon wafers and subsequently dried. Finally, the samples were coated with gold for 60 s and observed by SEM (JSM-IT810, Tokyo, Japan).

**Transmission Electron Microscopy (TEM) and Energy Dispersive Spectrometer (EDS).** The 100ng/μL TfR/PDL1-GBS@ZnO were adsorbed on carbon-coated copper grids for 10 minutes, washed twice with PBS, stained with 1% phosphotungstic acid for 10 min, and washed again with PBS. Dry under a white woven lamp overnight, the samples were observed on a JEOL-3010 transmission electron microscope (JSM-IT810, Tokyo, Japan).

**Cell Viability Assay.** To evaluate tumor cells killing by TfR/PDL1-GBS@ZnO in vitro, cell viability was assessed in 4T1 cells. Cells were seeded into 48-well plates at a density of 1×10^4^ cells/well and allowed to adhere overnight. The following day, cells were treated with different groups at 20 μg/mL for 24 h. Cell viability was measured using a Cell Counting Kit-8 following the manufacturer's instructions. After 24 h incubation, 20 μL of CCK8 (APE bio, Cell Counting Kit-8) was added into each well. Following incubation for 1 h at 37 ℃, the plates were measured for absorbance at 450 nm using a SpectraMax Microplate reader.

**Tumor cells and Immune cells co-culture.** Naïve T cells were isolated from spleen of BALB/c mice and cultured in RPMI-1640 supplemented with 10% FBS. BMDCs were prepared as described above. 4T1 cells were seeded in 48-well plate. 4T1 cells were treated with TfR/PDL1-GBS@ZnO (20 μg/mL) for 12 h and then replaced with fresh medium. Subsequently, DCs and T cells were added to the 4T1 cells at a 1:1:1 ratio. After 48h co-cultutre, the T cells were collected and analysis by flow cytometry.

**Annexin V/PI staining analysis apoptosis of tumor cells.** Cells were seeded in 12-well plate, the medium was changed and incubated with 20μg/mL TfR/PDL1-GBS@ZnO for different hours in the incubator. Cells were detached using trypsin to generate single-cell suspensions. Next, cells were dispersed in staining buffer and incubated with 2 μL L of Annexin V-FITC. 10 μL of PI was then added, for incubation in dark for 20 min. Then cells were immediately analyzed by flow cytometry.

**
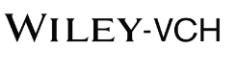
SUPPORTING INFORMATION**


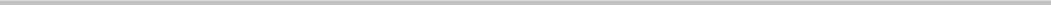


**ELISA kit assay.** The supernatant of corresponding samples was collected and measured with IFN- β, IFN-γ and TNF-α ELISA kit according to the manufacturer’s instructions.

**qRT-PCR.** 4T1 cells were passage into a 6-well plate and grown to ∼80% confluence within 24 h. The medium was changed and the cells were incubated with different groups. Cells were suspended, and RNA was extracted using *Evo M-MLV*RT Mix Kit (Accurate Bio) Extracted RNA was analyzed using SYBR Green Premix Pro Taq HS qPCR Kit (Accurate Bio). The primer sequences are listed as follows. m IFNβ-F: 5-ATAAGCAGCTCCAGCTCCAAG-3, m IFNβ-R: 5-ATCTCTTGGATGGCAAAGGCA-3, m CXCL10-F: 5-AAGTGCTGCCGTCATTTTCT-3, m CXCL10-R: 5-GTGGCAATGATCTCAACACG-3, m GAPDH-F: 5-CTGCCACCCAGAAGACTGTG-3, m GAPDH-R: 5-GGTCCTCAGTGTAGCCCAAG-3

**Patient-derived tumor-like cell cluster (PTC) models.** Tumor samples were obtained as a gift from Xiangtan Central Hospital and processed to establish patient-derived tumor-like cell cluster (PTC) models following previously reported protocols^[1]^. Briefly, tumor tissues were washed five times with PBS to thoroughly remove necrotic regions and adipose tissue. The tissues were then minced into small fragments and digested in 5 mL PBS containing 200 U/mL collagenase I and IV (Solarbio) at 37 °C for 1 h. During digestion, the samples were gently pipetted every 15 min to facilitate the generation of single cells. The digested cell suspension was filtered through a 70 μm cell strainer, followed by centrifugation at 300 × g for 10 min. The cell pellet was resuspended in PTC growth medium (gx-health, GX-KC1002M). The medium was replaced every 2–3 days. After 7–14 days of culture, tumor cell clusters were formed and subjected to subsequent treatment. The PTC was incubated with 20 μg/mL TfR/PDL1-GBS@ZnO for 48 hours. Then, samples were stained by immunofluorescence and analyzed by CLSM (Carl Zeiss LSM980, Jena, Germany) for 3D reconstruction.

**Quantitative proteomics analysis.** 4T1 cells were seeded in 10 cm cell culture flash (~80% confluency) and 20 μg/mL TfR/PDL1-GBS@ZnO incubated 4T1 cells for 24 h at 37 ^o^C. After that, cells were wash with cold DPBS for three times and cells were detached using trypsin to generate single-cell suspensions. The sample preparation procedure for quantitative proteomics analysis was performed by using a commercially available iST Sample Preparation kit (PreOmics, Germany) according to the provided protocols.

Briefly, 50 μL of Lyse buffer was added and heated at 95 °C for 10 minutes while agitating at 1000 rpm. After cooling the sample to room temperature, trypsin digestion buffer was added, and the mixture was incubated at 37 °C for 2 hours with agitation at 500 rpm. The digestion was halted using a stop buffer. Then, cleanup and desalination steps were conducted using the iST cartridge.

**
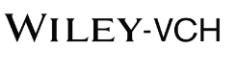
SUPPORTING INFORMATION**


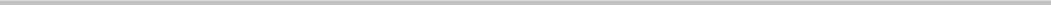


**In vivo therapeutic analysis.** For in vivo biodistribution analysis, the 4T1 tumor-bearing mice were tail-vein injected with Cy5-labeled DNA nanodevice, respectively. The in vivo imaging was conducted under an IVIS imaging system at different time intervals after administration. Then the mice were sacrificed immediately, the tumors and major organs were collected finally for ex vivo imaging.

Cytokine secretion. Tumor tissues were collected and weighed after treatment. The tissues were homogenized in ice-cold PBS and then centrifuged at 12,000 × g for 15 min at 4 °C. The supernatants were collected for IFN-γ measurement. Tumor levels of IFN-γ of mice from each group were measured with ELISA kit according to the manufacturer’s instructions.

Flow cytometry analysis assay. For Tumor-draining lymph nodes, The TDLN was collected, the tissues were then gently minced and pressed to release cells, and the resulting mixtures were filtered through a 75 μm nylon mesh to obtain single-cell suspensions. For tumor tissue, the tumor was first cut into small pieces, then were treated with 1× Triple Enzyme Mix at 37 °C for 2 h. The resultant mixtures were filtered through a 75 μm nylon filter to obtain single-cell suspensions. The cells were stained with flow antibody and then analyzed with flow cytometry.

Tumor Fixation and slice. Tumor tissues were harvested, rinsed briefly with PBS, and fixed in 4% paraformaldehyde at 4 °C for 24 h. After fixation, samples were dehydrated through a graded ethanol series, cleared in xylene, and infiltrated with molten paraffin. Finally, tissues were embedded in fresh paraffin blocks and cooled at room temperature before sectioning. After slicing, let it dry, followed by Immuno fluorescence, H&E staining and TUNEL.

hematological safety evaluation. Blood was collected by retro-orbital bleeding and placed at room temperature for 2 h, and then the serum was obtained by centrifuging the blood at 3000 rpm for 10 min. Serum levels of AST/ALT of mice from each group were measured with ELISA kit according to the manufacturer’s instructions. Serum levels of BUN of mice from each group were measured with Urea (BUN) colorimetric kit according to the manufacturer’s instructions.

**
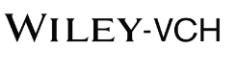
SUPPORTING INFORMATION**


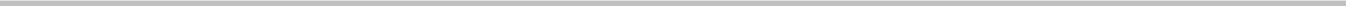


**Table S1**. List of DNA sequences used in this study (The TfR/PD-L1/EGFR^[2, 3]^ aptamer was marked in blue, cGAS activation sequence-GBS^[4, 5]^ was marked in red)

| Name | Sequence (5’-3’) |
| --- | --- |
| Primer-TfR | GGATAGGGATTCTGTTGGTCGGCTGGTTGGTATCCCCAGCTGGTATCTAGTTGAGCTGTCTAAC |
| Template-TfR-GBS | PHO-AACTAGATACCAGCTGGTATCCTTCAACGTCTAACGGATACCAACCAGCCGACCAACAGAATCCCTATCCCCCGAACTCCAGCAGGACCATTGCCCGTTAGACAGCTC |
| Primer-PDL1 | ACGGGCCACATCAACTCATTGATAGACAATGCGTCCACTGCCCGTCTGGTATCTAGTTGAGCTGTCTAAC |
| Template-PDL1-GBS | PHO-AACTAGATACCAGCTGGTATCCTTCAACGTCTAACACGGGCAGTGGACGCATTGTCTATCAATGAGTTGATGTGGCCCGTCCCCAATGGTCCTGCTGGAGTTCCCCGTTAGACAGCTC |
| Primer-1-GBS | CCAGCTGGTATCTAGTT GAGCTGTCTAAC |
| Template-1-GBS | PHO-AACTAGATACCAGCTGGTATCCTTCAACGTCTAACCCCGAACTCCAGCAGGACCATTGCCCGTTAGACAGCTC |
| Primer-2-GBS | CTGGTATCTAGTTGAGCTGTCTAAC |
| Template-2-GBS | PHO-AACTAGATACCAGCTGGTATCCTTCAACGTCTAACCCCCAATGGTCCTGCTGGAGTTCCCCGTTAGACAGCTC |
| Template-TfR-RS | PHO- AACTAGATACCAGCTGGTATCCTTCAACGTCTAACGGATACCAACCAGCCGACCAACAGAATCCCTATCCTTTTTTTTTTTGTACCGTACGTTAGACAGCTC |
| Template-PDL1-RS | PHO- AACTAGATACCAGCTGGTATCCTTCAACGTCTAACACGGGCAGTGGACGCATTGTCTATCAATGAGTTGATGTGGCCCGTGTACGGTACAAAAAAAAAAAGTTAGACAGCTC |
| TfR-apt | TGACTGATTTACGGGATAGGGATTCTGTTGGTCGGCTGGTTGGTATCC |
| PDL1-apt | CGTAAATCAGTCAACGGGCCACATCAACTCATTGATAGACAATGCGTCCACTGCCCGT |
| EGFR-apt | CGTAAATCAGTCAATGCTCAGTGCCGTTTCTTCTCTTTCGCTTTTTTTGCTTCTGAGCAT |
| Primer EGFR | ATGCTCAGTGCCGTTTCTTCTCTTTCGCTTTTTTTGCTTCTGAGCATCTGGTATCTAGTTGAGCTGTCTAAC |
| Template EGFR | PHO-AACTAGATACCAGCTGGTATCCTTCAACGTCTAACACGGGCAGTGGACGCATTGTCTATCAATGAGTTGATGTGGCCCGTCCCCAATGGTCCTGCTGGAGTTCCCCGTTAGACAGCTC |
| Template-EGFR-RS | PHO-AACTAGATACCAGCTGGTATCCTTCAACGTCTAACATGCTCAGAAGAAAAAAAGCGAAAGAGAAGAAACGGCACTGAGCATGTACGGTACAAAAAAAAAAAAAAAGTTAGACAGCTC |

**
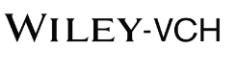
SUPPORTING INFORMATION**


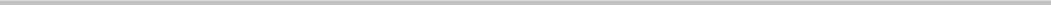


**Results and Discussion**


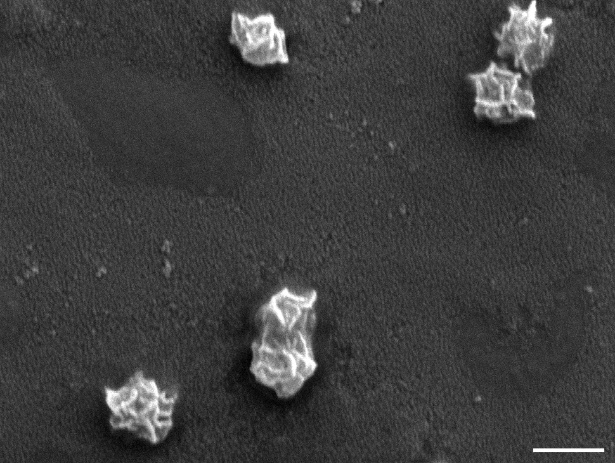


**Figure S1**. SEM image of the TfR/PDL1-GBS@ZnO. Scale bar = 200 nm.

**
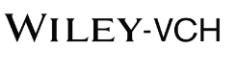
SUPPORTING INFORMATION**


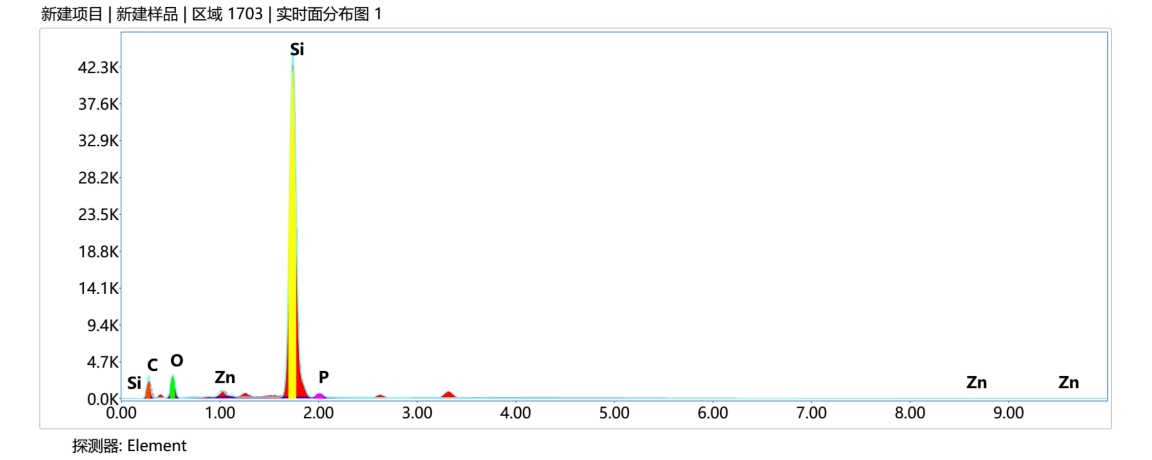

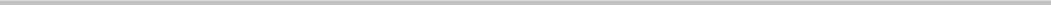


**Figure S2**. EDS analysis of TfR/PDL1-GBS@ZnO.

**
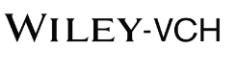
SUPPORTING INFORMATION**


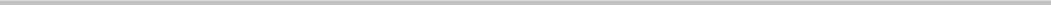


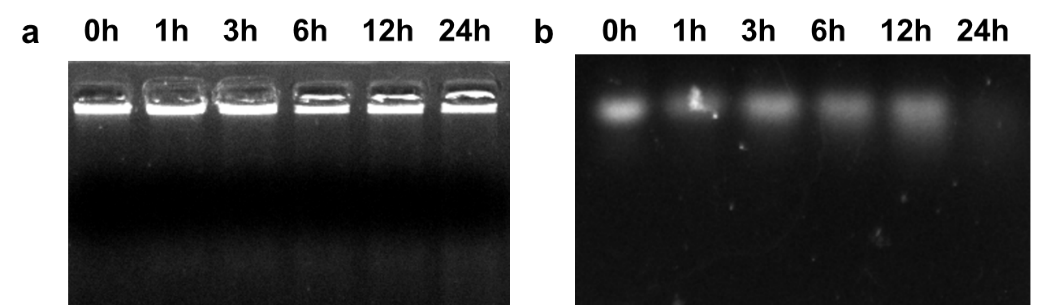


**Figure S3**. Gel analysis of TfR/PDL1-GBS@ZnO a) and TfR/PDL1-apt b) serum stability after incubation in 10% FBS at different time points.

**
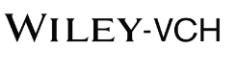
SUPPORTING INFORMATION**


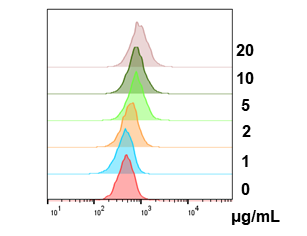

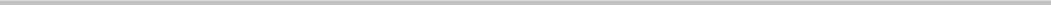


**Figure S4**. Flow cytometry analysis of TfR/PDL1-GBS@ZnO endocytosis with different dose in 4T1 cells.

**
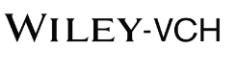
SUPPORTING INFORMATION**


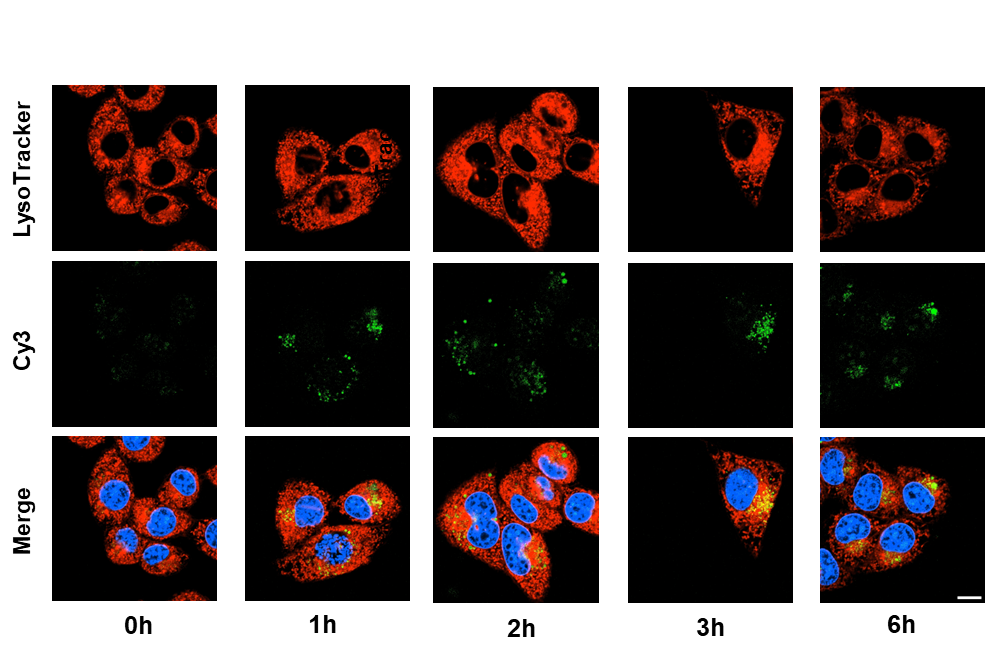

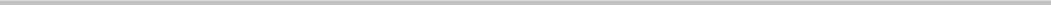


**Figure S5**. Confocal fluorescence image analysis of TfR/PDL1-GBS@ZnO internalization at different time points. Scale bar = 10 μm.

**
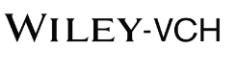
SUPPORTING INFORMATION**


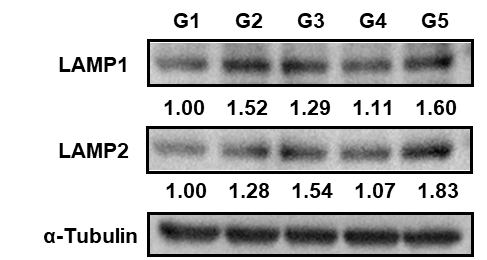

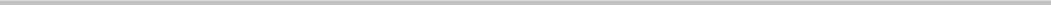


**Figure S6**. Western blot analysis of LAMP1/LAMP2 expression treated with different groups. G1: Blank, G2: TfR-GBS@ZnO, G3: PDL1-GBS@ZnO, G4: TfR/PDL1@ZnO, G5: TfR/PDL1-GBS@ZnO.

**
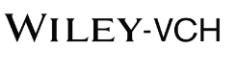
SUPPORTING INFORMATION**


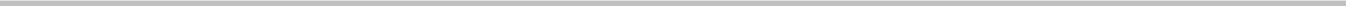


**
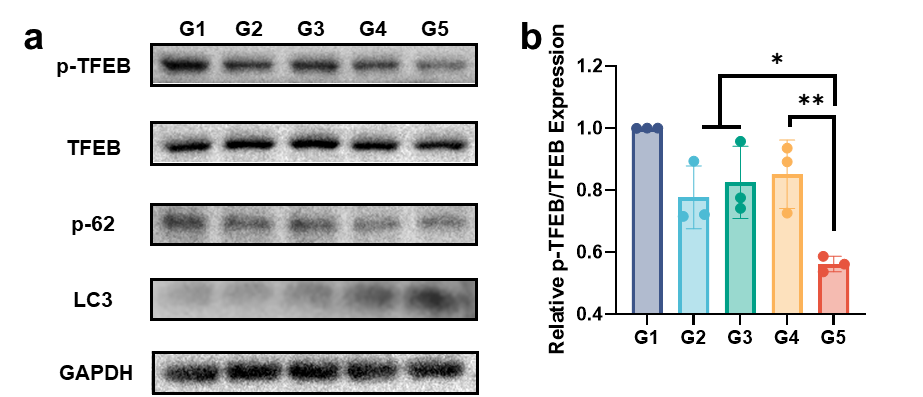
**

**Figure S7**. a) Western blot analysis of p-TFEB, TFEB, p-62 and LC3 expression treated with different groups. b) Statistical analysis of p-TFEB/TFEB treated with different groups. n=3, Data are presented as mean ± S.D., Significance (one-way ANOVA) is labeled above the data, *p < 0.05, **p < 0.01. G1: Blank, G2: TfR-GBS@ZnO, G3: PDL1-GBS@ZnO, G4: TfR/PDL1@ZnO, G5: TfR/PDL1-GBS@ZnO.

**
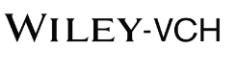
SUPPORTING INFORMATION**

**
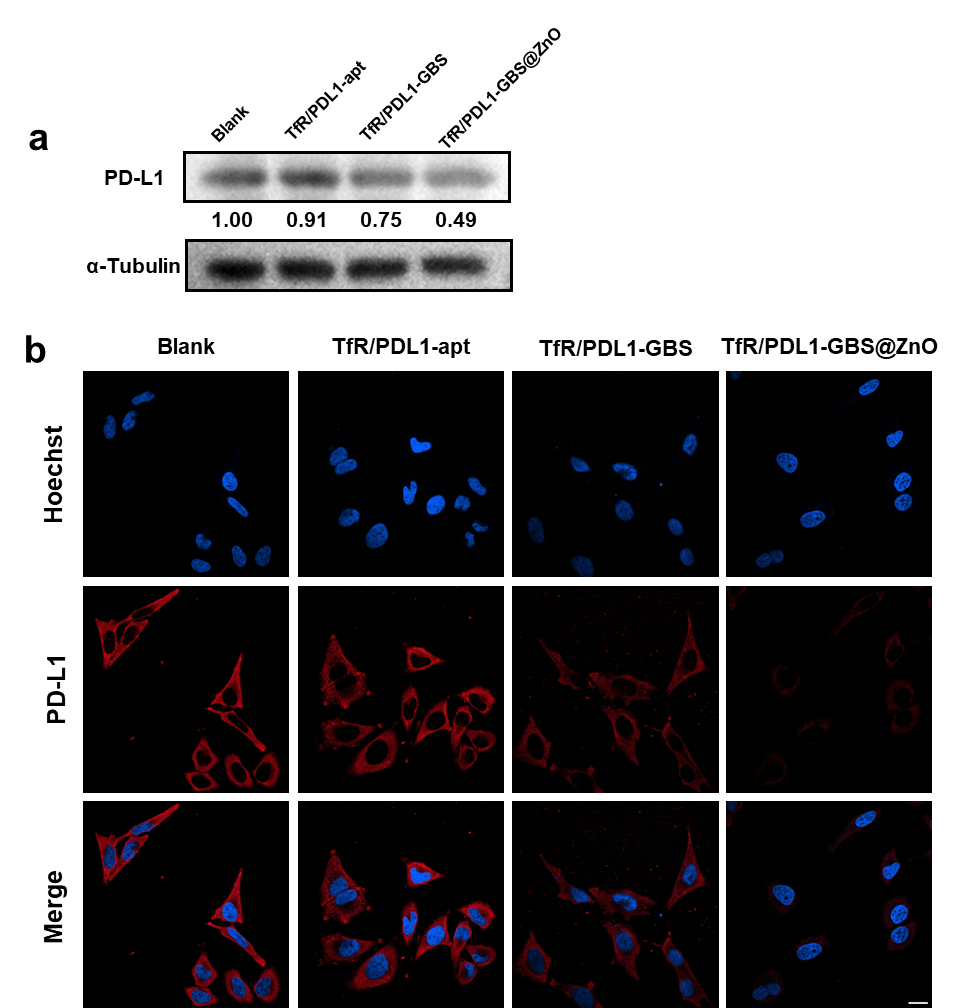
**
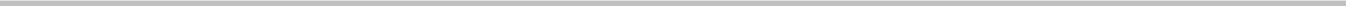


**Figure S8**. Western blot a) and Immunofluorescence b) analysis of PD-L1 degradation in 4T1 cells treated with TfR/PDL1-aptamer, TfR/PDL1-GBS and TfR/PDL1-GBS@ZnO. Scale bar = 10 μm.

**
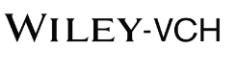
SUPPORTING INFORMATION**


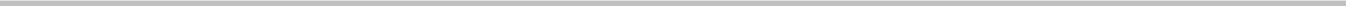


**
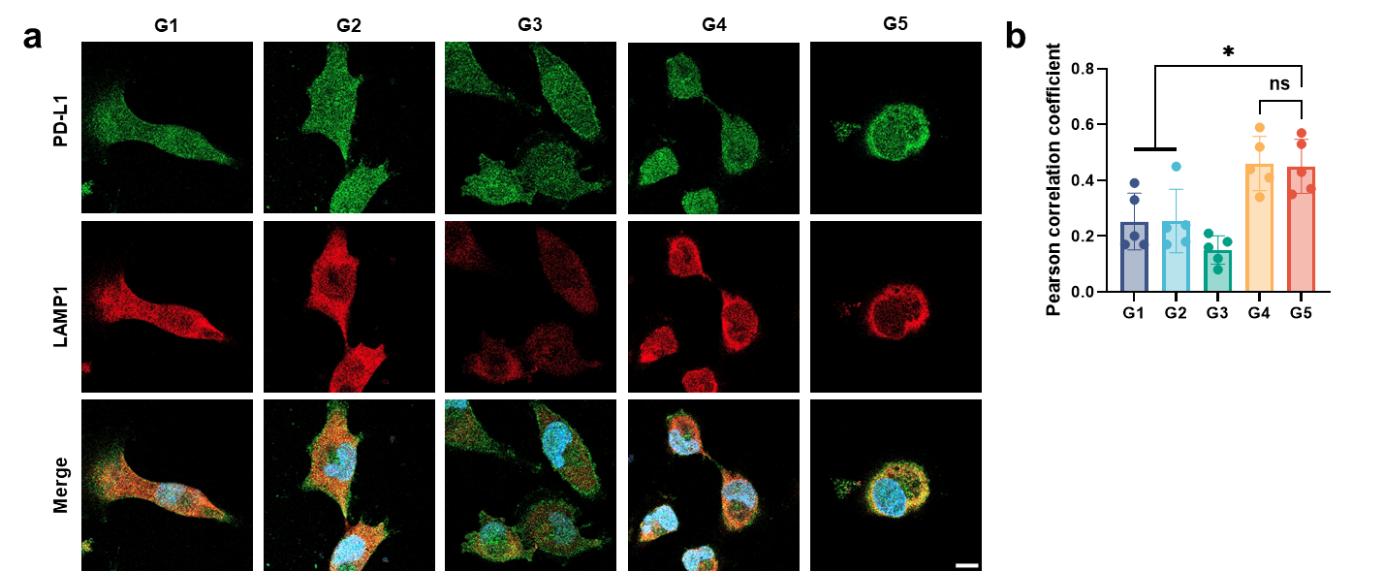
**

**Figure S9**. a) Immunofluorescence analysis of PD-L1 and LAMP1 colocalization in 4T1 cells treated with different groups. Scale bar = 10 μm. b) statistical quantification of Pearson correlation coefficient between PD-L1 and LAMP1, n = 5, Data are presented as mean ± S.D., Significance (one-way ANOVA) is labeled above the data, *p < 0.05. G1: Blank, G2: TfR-GBS@ZnO, G3: PDL1-GBS@ZnO, G4: TfR/PDL1@ZnO, G5: TfR/PDL1-GBS@ZnO.

**
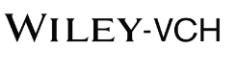
SUPPORTING INFORMATION**


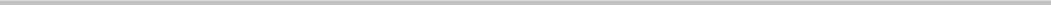
**
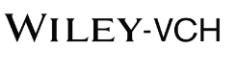
**

**
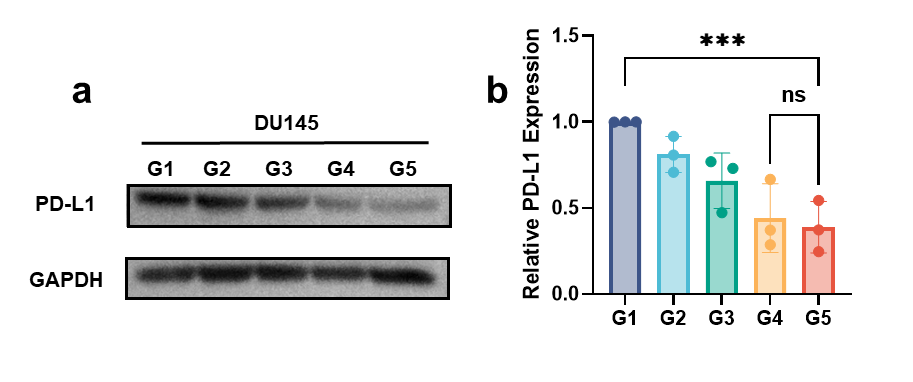
**

**Figure S10**. Western blot a) and statistical b) analysis of PD-L1 degradation in DU145 cells treated with different groups. n=3, Data are presented as mean ± S.D., Significance (one-way ANOVA) is labeled above the data, ***p < 0.001. G1: Blank, G2: TfR-GBS@ZnO, G3: PDL1-GBS@ZnO, G4: TfR/PDL1@ZnO, G5: TfR/PDL1-GBS@ZnO.

**
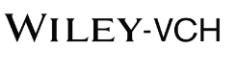
SUPPORTING INFORMATION**


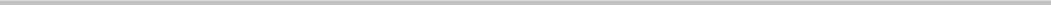

**Figure S11**. Western blot and statistical quantification of relative PD-L1 degradation after treatment with TfR/PDL1-GBS@ZnO. for the indicated times, The half-life of PD-L1 was determined by nonlinear regression analysis (t₁/₂ = 2.79 h, 95% CI: 1.38–5.95 h).

**
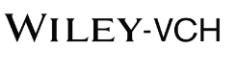
SUPPORTING INFORMATION**

**
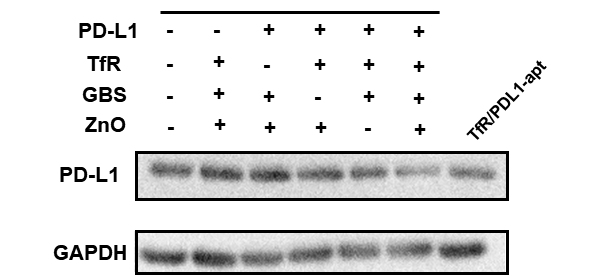
**
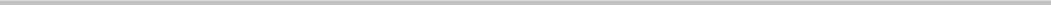
**
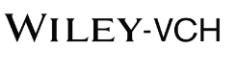
**

**Figure S12**. Western blot analysis of PD-L1 degradation in A549 cells treated with different groups.

**
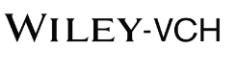
SUPPORTING INFORMATION**


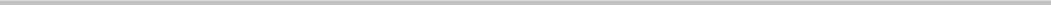
**
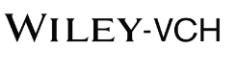
**

**
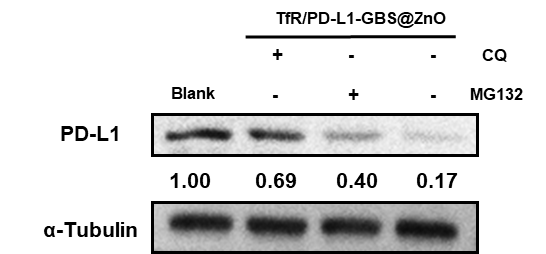
**

**Figure S13**. TfR/PDL1-GBS@ZnO-mediated membrane protein degradation in 4T1 cells treated with CQ and MG132.

**
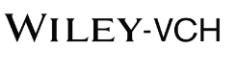
SUPPORTING INFORMATION**

**
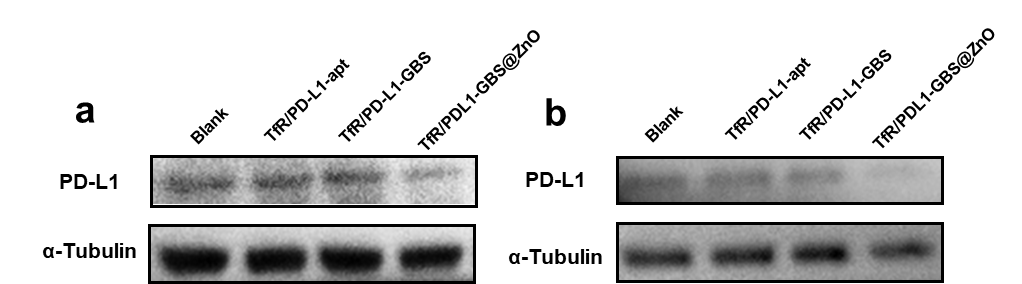
**
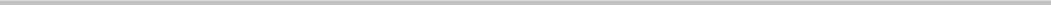
**
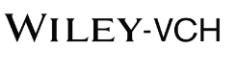
**

**Figure S14**. Western blot analysis of PD-L1 degradation in human breat cancer MDA-MB-231 cells and Hela cells treated with TfR/PDL1-apt, TfR/PDL1-GBS and TfR/PDL1-GBS@ZnO.

**
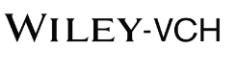
SUPPORTING INFORMATION**

**
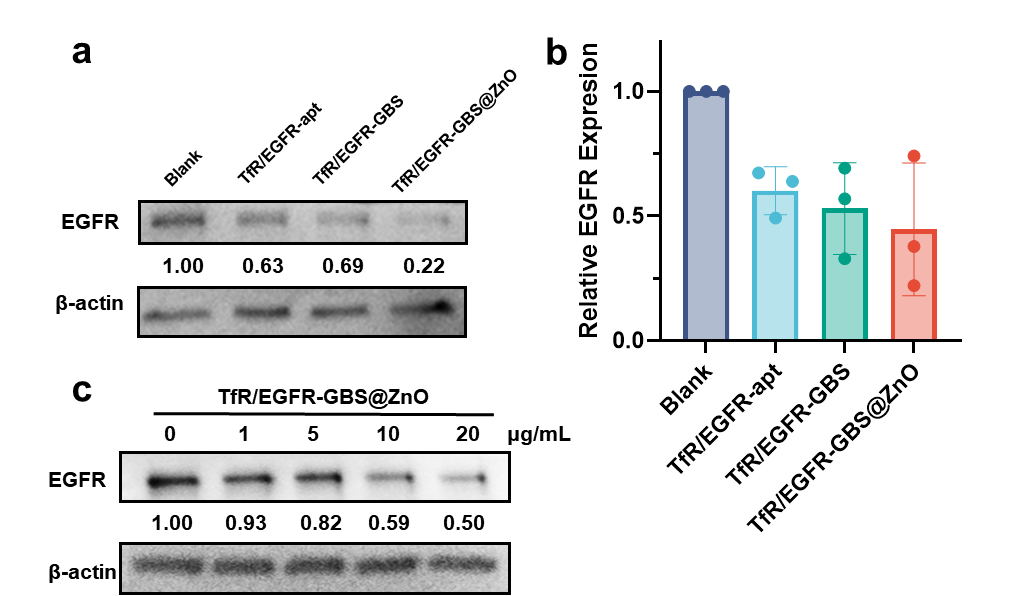
**
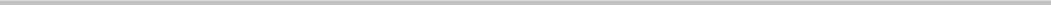
**
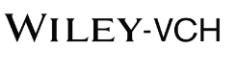
**

**Figure S15**. Western blot a) and Statistical analysis b) of EGFR degradation in A549 cells treated with different groups. c) Western blot analysis of EGFR degradation in A549 cells treated with different TfR/EGFR-GBS@ZnO dose. n=3.

**
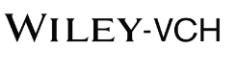
SUPPORTING INFORMATION**


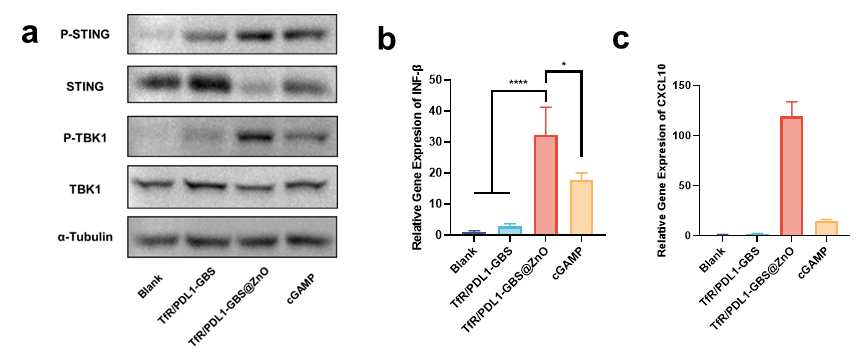

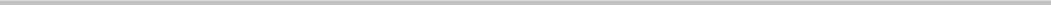
**
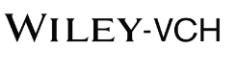
**

**Figure S16.** a) Western blot analysis of STING pathway activation in 4T1 cells treated with TfR/PDL1-GBS, TfR/PDL1-GBS@ZnO and cGAMP. mRNA expression of INF-β b) and CXCL10 c) after different treatment in 4T1 cells. n=3, Data are presented as mean ± S.D., Significance (one-way ANOVA) is labeled above the data, *p < 0.05, ****p < 0.0001.

**
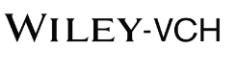
SUPPORTING INFORMATION**


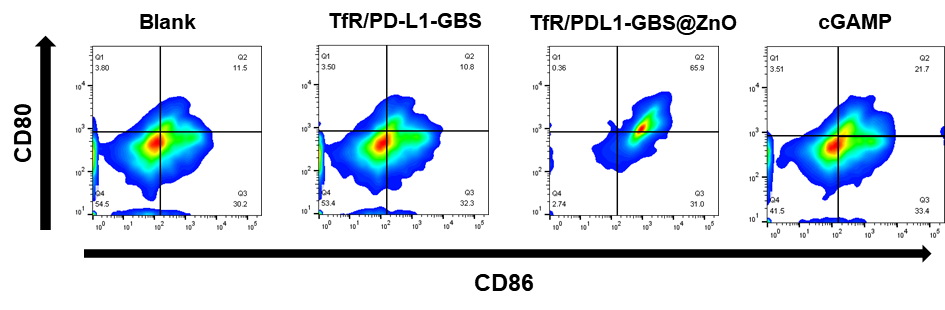

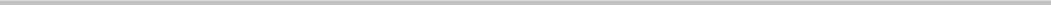
**
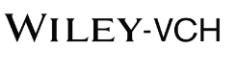
**

**Figure S17.** The flow cytometric analysis of BMDCs maturation treated with TfR/PDL1-GBS, TfR/PDL1-GBS@ZnO and cGAMP.

**
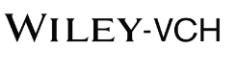
SUPPORTING INFORMATION**


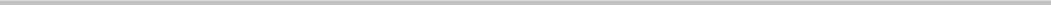
**
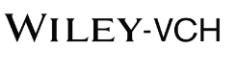
**

**
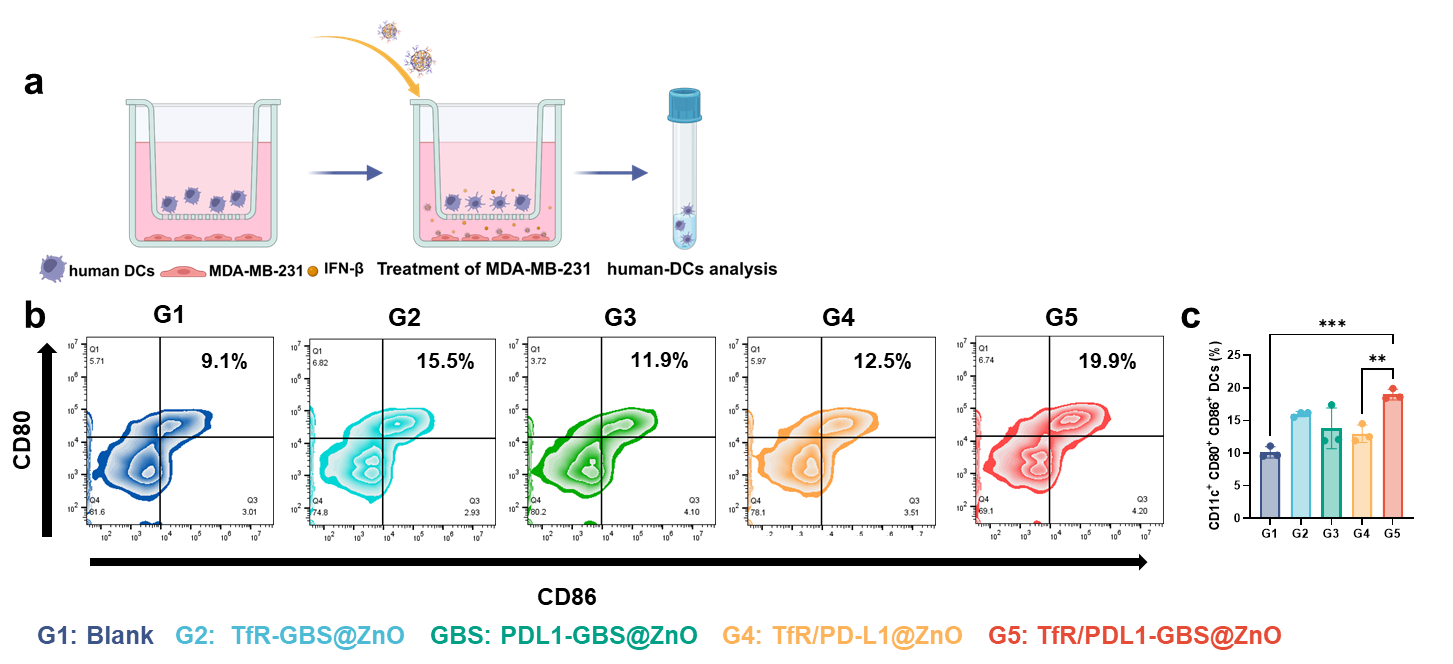
**

**Figure S18.** a) Schematic illustration of in vitro assessment of human DCs maturation. The upper layer is human DCs and the lower layer is MDA-MB-231 cells. Created in BioRender.com. Flow cytometry b) and statistical analysis c) of human-DCs maturation after different treatments. Data are presented as mean ± S.D., n = 3, Significance (one-way ANOVA) is labeled above the data, **p < 0.01, ***p < 0.001.

**
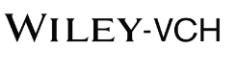
**

**
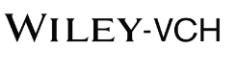
SUPPORTING INFORMATION**


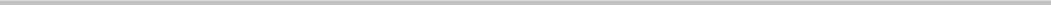


**Figure S19.** 4T1 cells viability treated with different groups. G2: TfR-GBS@ZnO, G3: PDL1-GBS@ZnO, G4: TfR/PDL1-RS@ZnO, G5: TfR/PDL1-GBS@ZnO. n=3.

**
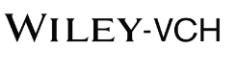
SUPPORTING INFORMATION**


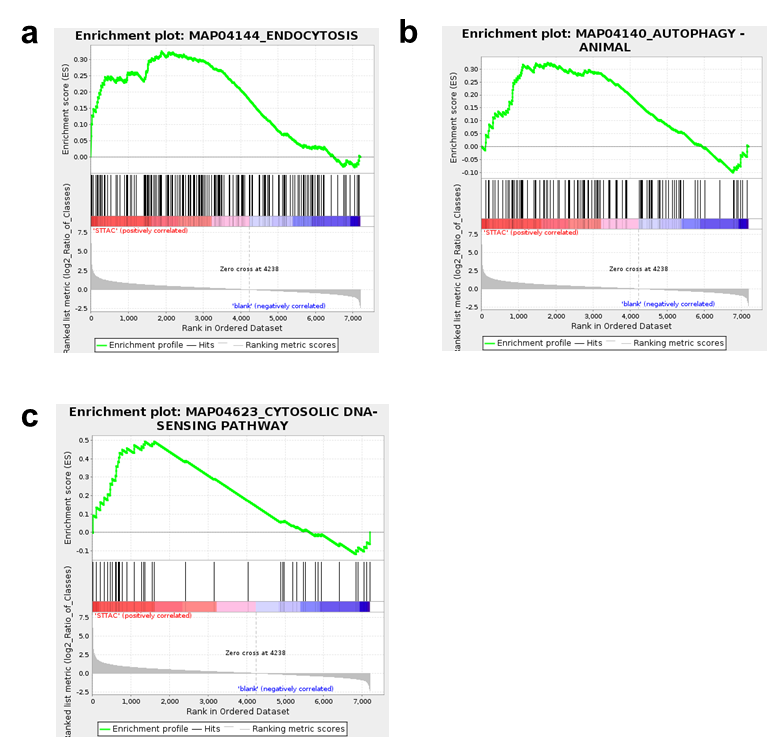

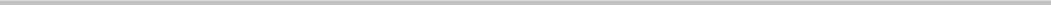


**Figure S20**. GSEA analysis of endocytosis a), autophagy b) and DNA sensing pathway c) related gene expression between the Blank and TfR/PDL1-GBS@ZnO groups.

**
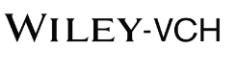
SUPPORTING INFORMATION**


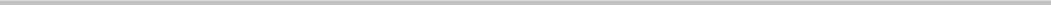
**
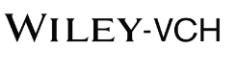
**

**
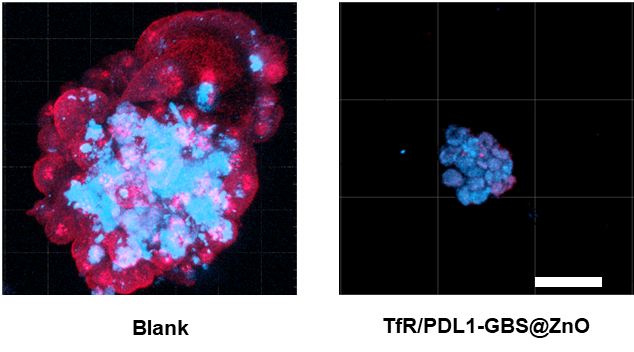
**

**Figure S21.** 3D immunofluorescence imaging of a PTC model from lung adenocarcinoma after TfR/PDL1-GBS@ZnO treatment, scale bar = 50 μm

**
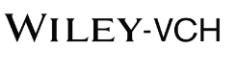
SUPPORTING INFORMATION**


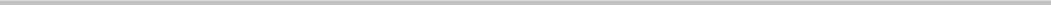


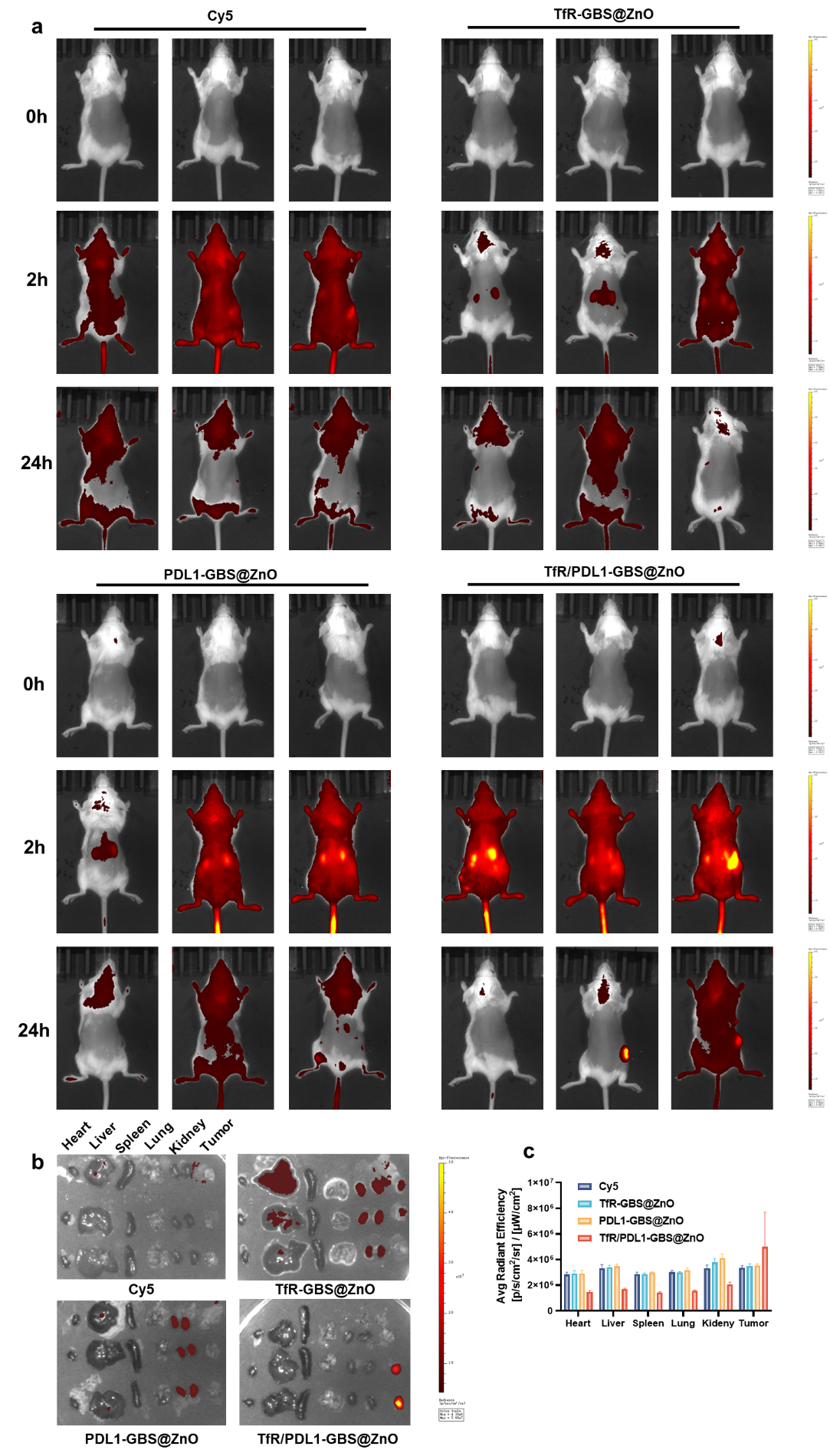


**Figure S22.** Evulation of organ distribution of TfR/PDL1-GBS@ZnO in vivo. a) The in vivo biodistribution of different nanodevices in tumor-bearing mice. b) Ex vivo imaging of tumor and othermajor tissues collected from mice treated with different nanodevices (2 mg/kg). c) Statistical analysis analysis of b). n=3.

**
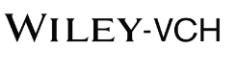
SUPPORTING INFORMATION**


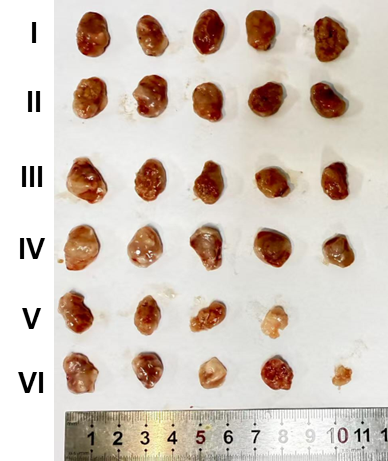

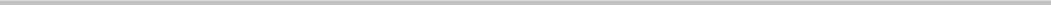


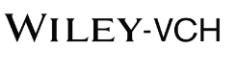


**Figure S23** Photograph of the dissected 4T1 tumors from mice treated with different groups. i. Blank, ii, TfR-GBS@ZnO, iii. PDL1-GBS@ZnO, iv. TfR/PDL1-RS@ZnO, v. TfR/PDL1-GBS@ZnO, vi. diABZI.
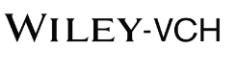
.

**
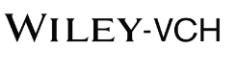
SUPPORTING INFORMATION**


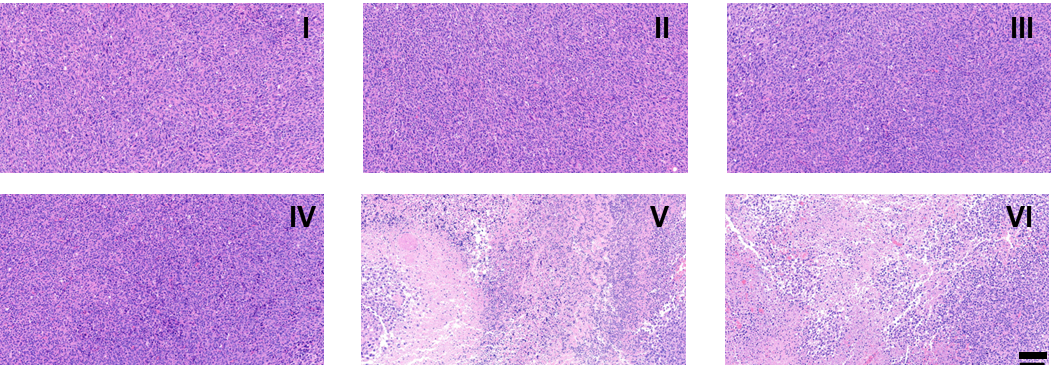

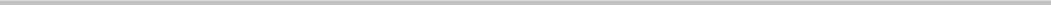


**Figure S24.** H&E analysis of tumors from different groups. i. Blank, ii, TfR-GBS@ZnO, iii. PDL1-GBS@ZnO, iv. TfR/PDL1-RS@ZnO, v. TfR/PDL1-GBS@ZnO, vi. diABZI. Scale bar = 100 μm.

**
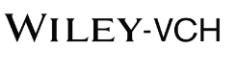
SUPPORTING INFORMATION**


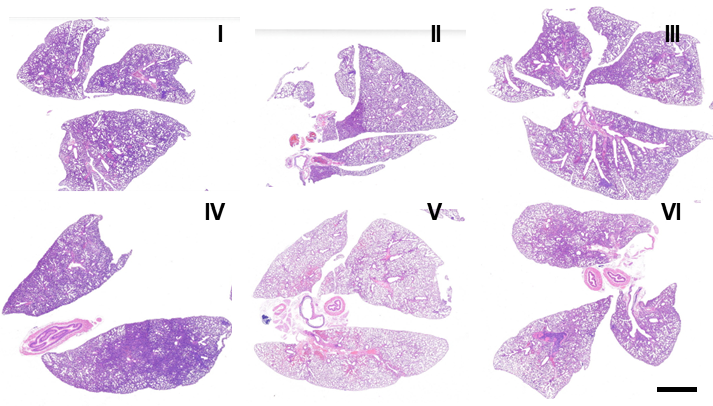

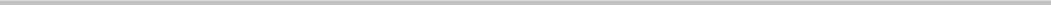


**Figure S25.** H&E analysis of lung with metastatic breast tumors from different groups. i. Blank, ii, TfR-GBS@ZnO, iii. PDL1-GBS@ZnO, iv. TfR/PDL1-RS@ZnO, v. TfR/PDL1-GBS@ZnO, vi. diABZI. Scale bar = 2000 μm.

**
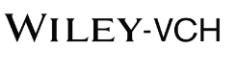
SUPPORTING INFORMATION**


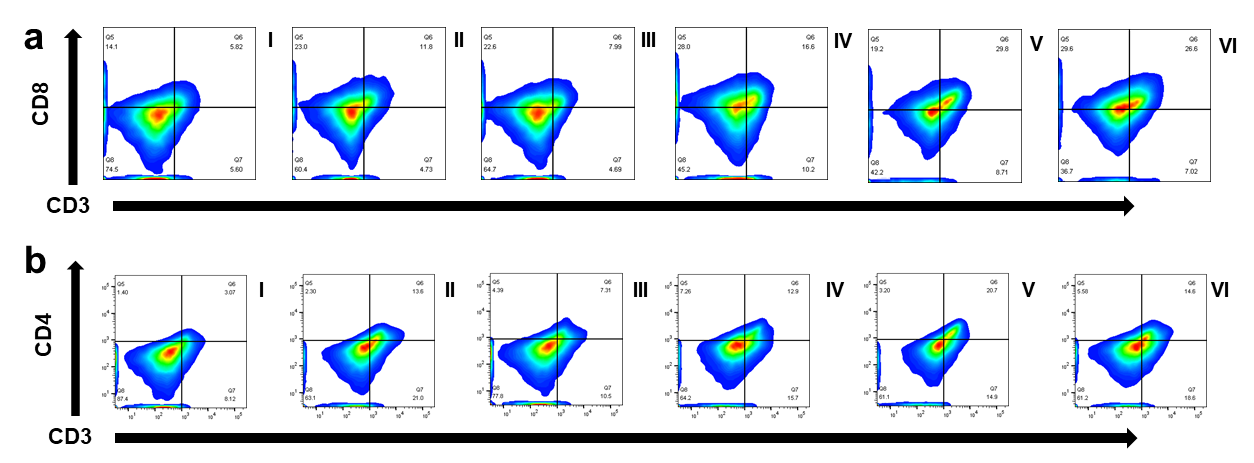

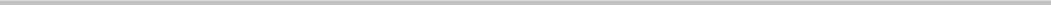


**Figure S26.** Flow cytometric analysis of CD8^+^ T cell a) and CD4^+^ T cell b) in the immune cell populations of tumor tissues treated with different groups. i. Blank, ii, TfR-GBS@ZnO, iii. PDL1-GBS@ZnO, iv. TfR/PDL1-RS@ZnO, v. TfR/PDL1-GBS@ZnO, vi. diABZI.
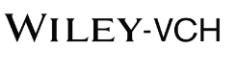


**
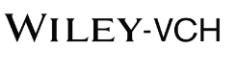
SUPPORTING INFORMATION**

**Figure S27.** Plasma concentration–time profiles of different treatment groups in mice. G1. Blank, G2. TfR-GBS@ZnO, G3. PDL1-GBS@ZnO, G4. TfR/PDL1-RS@ZnO, G5. TfR/PDL1-GBS@ZnO. n=3

**SUPPORTING INFORMATION**

**Figure S28.** Elisa analysis of ALT a) and AST b) percentage in tumor tissues of different groups. c) Urea (BUN) colorimetric assay analysis of BUN levels in mouse serum after treatment with different groups. n=5. G1. Blank, G2. TfR-GBS@ZnO, G3. PDL1-GBS@ZnO, G4. TfR/PDL1-RS@ZnO, G5. TfR/PDL1-GBS@ZnO.

**SUPPORTING INFORMATION**

**References**

[1] Y. Hou, H. L. Liang, X. Yu, et al., *Science Translational Medicine* **2021**, 13, eabb0130.38

[2] Y. Li, X. Liu, L. Yu, X. Huang, X. Wang, D. Han, Y. Yang, Z. Liu, *Journal of the American Chemical Society* **2023**;

[3] N. Zhang, T. Bing, L. Shen, L. Feng, X. Liu, D. Shangguan, *Int J Mol Sci* **2021**, 22.

[4] Y. Chen, R. Li, Q. Duan, et al., *Advanced. Science.***2024**, 11, 2400149.

[5] A.-M. Herzner, C. A. Hagmann, M. Goldeck, S. Wolter, K. Kübler, S. Wittmann, T. Gramberg, L. Andreeva, K.-P. Hopfner, C. Mertens, T. Zillinger, T. Jin, T. S. Xiao, E. Bartok, C. Coch, D. Ackermann, V. Hornung, J. Ludwig, W. Barchet, G. Hartmann, M. Schlee, *Nature Immunology* **2015**, 16, 1025-1033.
